# Simultaneous failure of two sex-allocation invariants: implications for sex-ratio variation within and between populations

**DOI:** 10.1098/rspb.2015.0570

**Published:** 2015-07-07

**Authors:** António M. M. Rodrigues, Andy Gardner

**Affiliations:** 1 Department of Zoology, University of Cambridge, Downing Street, Cambridge CB2 3EJ, UK; 2 Wolfson College, Barton Road, Cambridge CB3 9BB, UK; 3 School of Biology, University of St Andrews, Dyers Brae, St Andrews KY16 9TH, UK

**Keywords:** constant male hypothesis, dispersal, kin selection, local mate competition, local resource competition, viscosity

## Abstract

Local mate competition (LMC) occurs when male relatives compete for mating opportunities, and this may favour the evolution of female-biased sex allocation. LMC theory is among the most well developed and empirically supported topics in behavioural ecology, clarifies links between kin selection, group selection and game theory, and provides among the best quantitative evidence for Darwinian adaptation in the natural world. Two striking invariants arise from this body of work: the number of sons produced by each female is independent of both female fecundity and also the rate of female dispersal. Both of these invariants have stimulated a great deal of theoretical and empirical research. Here, we show that both of these invariants break down when variation in female fecundity and limited female dispersal are considered in conjunction. Specifically, limited dispersal of females following mating leads to local resource competition (LRC) between female relatives for breeding opportunities, and the daughters of high-fecundity mothers experience such LRC more strongly than do those of low-fecundity mothers. Accordingly, high-fecundity mothers are favoured to invest relatively more in sons, while low-fecundity mothers are favoured to invest relatively more in daughters, and the overall sex ratio of the population sex ratio becomes more female biased as a result.

## 1. Introduction

Local mate competition (LMC) occurs when male relatives compete for mating opportunities, and this may favour the evolution of female-biased sex allocation [1]. Hamilton's [2] classic study of LMC helped to clarify the relationship between kin selection and group selection and provided an early application of game-theoretic thinking to evolutionary biology. Hamilton's model considered a scenario in which one or more mothers each contribute a fixed number of offspring to a patch, followed by random mating among the offspring in the patch and complete dispersal of mated females to new patches. This basic scenario has subsequently been extended in multiple directions, and the theory of LMC has come to be one of the most successful topics in behavioural ecology, boasting a healthy interplay of theory and empiricism and an impressive fit between mathematical prediction and real-world observation, and providing among the best quantitative support for Darwinian adaptation in the natural world (reviewed by West [1]).

Two striking invariance results have emerged from the study of LMC, both of which concern constancy in the number of sons produced by mothers, despite the relaxation of key assumptions in Hamilton's [2] model. The first invariant arises in the context of relaxing the assumption that all mothers have the same fecundity. When fecundity varies, the sons of high-fecundity mothers experience relatively more-intense LMC and, accordingly, the proportion of a mother's reproductive resources that she invests into sons is expected to be inversely proportional to her fecundity. Consequently, the absolute number of sons that she produces is expected to be independent of her fecundity [3–5]. This result has been termed the ‘Constant Male Hypothesis’ (CMH) [6] and has stimulated a great deal of theoretical and empirical work [3–21].

The second invariant arises in the context of relaxing the assumption of complete female dispersal following mating. When female dispersal is incomplete, the associated increase in relatedness within mating groups enhances LMC among males (promoting female bias) but this effect is offset by an increase in local resource competition (LRC; [1]) among related females (inhibiting female bias), such that the number of sons that a mother produces is expected to be independent of the rate of female dispersal [22,23]. This surprising result has stimulated huge interest in the interplay of relatedness and kin competition in driving the evolution of social evolution in so-called ‘viscous populations’ [24–44].

Here, we consider the scenario in which there is both variation in female fecundity and also limited dispersal of females following mating. We develop a kin-selection model of sex allocation in the context of a population that is both class-structured—in terms of individuals being separated into males versus females, and breeding females being separated into high- and low-fecundity mothers—and also genetically structured—as a consequence of limited dispersal of females between patches from generation to generation. We derive analytical results to explore how the unbeatable sex-allocation strategy varies as a function of inequality in female fecundity and the rate of female dispersal, both in terms of an individual female's production of sons and also in terms of the population average investment into males, when sex allocation is either facultatively adjusted according to female condition or else obligately fixed at a value that balances the selective pressures faced by high- and low-fecundity mothers.

## 2. Results and discussion

### (a) Mathematical model

We assume an infinite island model [45], in which each patch contains one high-fecundity mother (denoted by the subscript H) and one low-fecundity mother (denoted by the subscript L). High-fecundity mothers produce a very large number *F*_H_ of offspring and low-fecundity mothers produce a large number *F*_L_ = (1 − *s*)*F*_H_ of offspring, where 0 ≤ *s* ≤ 1. Each mother is free to adjust the proportion of her offspring that are male versus female, with a focal high-fecundity mother producing *F*_H_ *z*_H_ sons and *F*_H_(1 − *z*_H_) daughters and a focal low-fecundity mother producing *F*_L_ *z*_L_ sons and *F*_L_(1 − *z*_L_) daughters, where 0 ≤ *z*_H_, *z*_L_ ≤ 1. After reproduction, mothers die, and offspring mate at random within their patch, with each female mating once. Following mating, males die, and each female attempts to disperse to a new patch at random with probability *d* or else remains in her natal patch with probability 1 − *d*. Dispersing females die on the way to their new patch with probability *k* and arrive safely at their new patch with probability 1 − *k*. Following dispersal, in each patch, one female is chosen at random to be the high-fecundity mother and one female is chosen at random to be the low-fecundity mother, with all other females dying, and this recovers the beginning of the life cycle. Table 1 provides a summary of key model notation.

**Table 1.** Summary of key model notation.

symbol	meaning
*c*	class-reproductive value
*d*	probability of dispersal
*φ*	probability of co-philopatry
*k*	cost of dispersal
*n*	relative number of juveniles
*r*	relatedness coefficients
*s*	reproductive inequality
*u*	frequency of juveniles
*z*	sex ratio

### (b) Evolution of sex allocation

Employing a neighbour-modulated fitness approach to kin selection [46–52], we find that the condition for natural selection to favour an increase in the sex allocation (i.e. investment into sons) of high-fecundity mothers is2.1
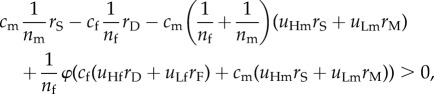
where: *c*_m_ is the class-reproductive value of males; *c*_f_ is the class-reproductive value of females; *n*_f_ = 1 − *z*_H_ + (1 − *z*_L_)(1 − *s*) is the relative number of juvenile females, and *n*_m_ = *z*_H_ + *z*_L_(1 − *s*) the relative number of juvenile males, produced in a local patch; *u*_Hf_ = (1 − *z*_H_)/*n*_f_ is the frequency of juvenile females who have high-fecundity mothers; *u*_Lf_ = ((1 − *z*_L_)(1 − *s*))/*n*_f_ is the frequency of juvenile females who have low-fecundity mothers; *u*_Hm_ = *z*_H_/*n*_m_ is the frequency of juvenile males who have high-fecundity mothers; *u*_Lm_ = (*z*_L_(1 − *s*))/*n*_m_ is the frequency of juvenile males who have low-fecundity mothers; *φ* = (1 − *d*)^2^/(1 − *kd*)^2^ is the probability that two random juvenile females in a patch following dispersal are both born in this patch (i.e. the probability of co-philopatry); *r*_D_ is the relatedness between a mother and her daughters; *r*_S_ is the relatedness between a mother and her sons; *r*_F_ is the relatedness between a mother and a juvenile female who is a daughter of the other mother; *r*_M_ is the relatedness between a mother and a juvenile male who is a son of the other mother. All of these relatedness coefficients are calculated from the mother's perspective (see electronic supplementary material for details).

A similar condition may be derived for low-fecundity mothers:2.2
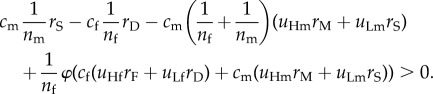


### (c) Rarer-sex effect

The first two terms on the left-hand side (LHS) of each of these conditions (2.1) and (2.2) jointly describe the rarer-sex effect [53]. In both conditions, the first term *c*_m_(1/*n*_m_)*r*_S_ describes the mother's marginal inclusive-fitness gain through increased allocation to her sons, holding fixed the mating success of each individual son, and this promotes an increased investment into sons. The second term −*c*_f_(1/*n*_f_)*r*_D_ describes her marginal inclusive-fitness loss through decreased allocation to her daughters, holding fixed the mating success of each individual daughter, and this inhibits an increased investment into sons.

Under haploid and diploid inheritance, a mother is equally related to her sons and her daughters (*r*_S_ = *r*_D_), and the class-reproductive value of males and females are equal (*c*_m_ = *c*_f_), so which of these two terms is larger depends on the relative number of males and females (*n*_m_ versus *n*_f_). In particular: when males are the rarer sex (*n*_m_ < *n*_f_), the marginal inclusive-fitness gain through sons outweighs the marginal inclusive-fitness loss through daughters, such that increased allocation to sons is favoured; and, likewise, an increased allocation to daughters is favoured when females are the rarer sex (*n*_m_ > *n*_f_). Under haplodiploid inheritance, a mother is twice as related to her sons as she is to her daughters in the context of outbreeding (*r*_S_ = 2*r*_D_), because while all of her son's genes derive from her, half of her daughter's genes derive from an unrelated male. But, as the class-reproductive value of females is twice that of males (*c*_f_ = 2*c*_m_), the rarer-sex effect once again favours increased in investment into offspring of whichever sex is rarer [2,54]. However, in the context of inbreeding, a mother is relatively more related to her daughters than to her sons (*r*_S_ < 2*r*_D_), because her daughter's paternal-origin genes come from a male with whom she shares genes in common and, consequently, a slight female bias is expected as a consequence of inbreeding *per se* [3,55].

### (d) Local mate competition

The third term in each condition is the LMC effect, which arises as a consequence of greater investment into sons leading to more competition among related males for fewer mating opportunities with females, to the extent that mating occurs among relatives [2]. Firstly, a greater investment into sons leads to an increase in the number of mate competitors [56]. The corresponding increase in competition depends on the number of males that are being produced by both mothers: if very few males are being produced, then an additional male has a large impact upon the mating success of other males; but if many males are already being produced, then an additional male has only a small impact. Accordingly, the amount of additional LMC falls when the number of male offspring rises, as captured by the factor 1/*n*_m_ in the LMC term. Secondly, a greater investment into sons leads to a decrease in the number of females for them to mate with [56]: if very few females are being produced, then one fewer female has a large impact upon the mating success of other males; but if many females are being produced, then one fewer female has only a small impact. Accordingly, the amount of additional LMC falls when the number of female offspring rises, as captured by the factor 1/*n*_f_ in the LMC term. Thirdly, the amount of LMC also depends on the value of males for mothers. This value depends on the reproductive value of males (*c*_m_), and on the relatedness between the mother and the males, which is *u*_Hm_*r*_S_ + *u*_Lm_*r*_M_ from the perspective of a high-fecundity mother, and *u*_Hm_*r*_M_ + *u*_Lm_*r*_S_ from the perspective of a low-fecundity mother. In viscous populations, mating is more likely to occur among closely related individuals, so both relatedness coefficients (i.e. *r*_S_ and *r*_M_) increase with population viscosity. This means that the intensity of LMC rises when population viscosity rises, as shown in electronic supplementary material, figure H1*a*,*c*). A high-fecundity mother is more related to her sons than she is to her patchmate's sons (i.e. *r*_S_ > *r*_M_), and this is exactly the same for a low-fecundity mother (i.e. *r*_S_ > *r*_M_). By contrast, a high-fecundity mother has a higher proportion of sons among the males than does a low-fecundity mother (i.e. *u*_Hm_ > *u*_Lm_). Accordingly, high-fecundity mothers suffer more LMC than do low-fecundity mothers, as shown in electronic supplementary material, figure H1*a*,*c*. This difference leads to the CMH result in the context of full female dispersal: each mother's proportional investment into sons is inversely proportional to her fecundity, so that her absolute investment into sons is constant, i.e. independent of her fecundity [3,5].

### (e) Local resource competition

The fourth term in each condition is the LRC effect, which arises as a consequence of greater investment into sons, leading to less competition among related females for breeding opportunities, to the extent that competition for breeding opportunities occurs among relatives [57]. Firstly, the benefit of the LRC effect depends on the number of females mothers produce (i.e. *n*_f_): if there are few females, one fewer female has a large impact on the reproductive success of other females; but if there are many females, one fewer female has only a small impact. Accordingly, the inclusive-fitness benefit of the LRC effect falls when the number of females rises, as captured by the factor 1/*n*_f_ in the LRC term. Secondly, two juveniles are related only if they are both locals, which occurs with probability *φ*. Thirdly, a mother values juvenile females according to the reproductive value of the latter (*c*_f_), and according to her relatedness to them, which is *u*_Hf_*r*_D_ + *u*_Lf_*r*_F_ from the perspective of a high-fecundity mother, and *u*_Hf_*r*_F_ + *u*_Lf_*r*_D_ from the perspective of a low-fecundity mother. Each mother is more related to her own daughters than she is to her patchmate's daughters (i.e. *r*_D_ > *r*_F_), but because a high-fecundity mother has a higher proportion of daughters among the juvenile females in her patch (i.e. *u*_Hf_ > *u*_Lf_), she is more related to the juvenile females than is the low-fecundity mother (i.e. *u*_Hf_*r*_D_ + *u*_Lf_*r*_F_ > *u*_Hf_*r*_F_ + *u*_Lf_*r*_D_). Fourthly, a mother values juvenile males according to the reproductive value of the latter (*c*_m_), and according to her relatedness to them, which is *u*_Hf_*r*_S_ + *u*_Lf_*r*_M_ from the perspective of a high-fecundity mother, and *u*_Hf_*r*_M_ + *u*_Lf_*r*_S_ from the perspective of a low-fecundity mother. Again, the high-fecundity mother is more related to the juvenile males than is the low-fecundity mother (i.e. *u*_Hf_*r*_S_ + *u*_Lf_*r*_M_ > *u*_Hf_*r*_M_ + *u*_Lf_*r*_S_). Accordingly, the high-fecundity mother gains more from the LRC effect than a low-fecundity mother, as shown in electronic supplementary material, figure H1*b*,*d*. As a consequence of this difference, high-fecundity mothers are relatively favoured to invest more into sons and low-fecundity mothers are relatively favoured to invest more into daughters.

### (f) Explicit demography

**Figure 1.**
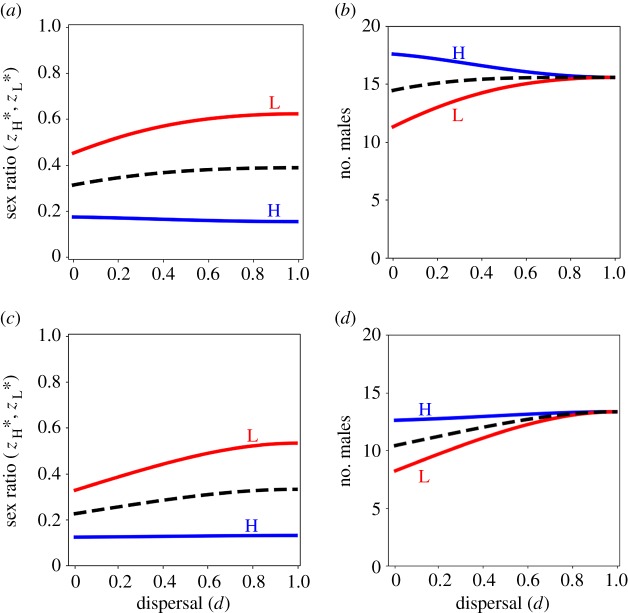
Facultative sex allocation for the basic model. (*a*,*c*) High-fecundity mothers (blue line, H) are favoured to produce relatively less female-biased sex ratios, whereas low-fecundity mothers (red line, L) are favoured to produce relatively more female-biased sex ratios, in viscous populations (*d* < 1), and the population average sex ratio strategy (dashed line) becomes more female-biased as the population becomes more viscous (lower *d*), under haploidy and diploidy (*a*), and under haplodiploidy (*c*). (*b*,*d*) High-fecundity mothers (blue line, H) are favoured to invest relatively more into sons than are low-fecundity mothers (red line, L) in viscous populations (*d* < 1), and the population average allocation to sons (dashed line) decreases as the population becomes more viscous (lower *d*), under haploidy and diploidy, and under haplodiploidy. However, while under haploidy and diploidy high-fecundity mothers are favoured to invest more into sons, under haplodiploidy high-fecundity mothers are favoured to invest less into sons, as the population becomes more viscous. We arbitrarily set the number of offspring of a high-fecundity mother to 100. Parameter values: *k* = 0 and *s* = 0.75.

We have expressed conditions (2.1) and (2.2) in terms of emergent quantities—such as relatedness and reproductive value—that do not take explicitly assumed values but rather may be derived from the model's assumptions and expressed in terms of its demographic parameters (see electronic supplementary material, appendix, for details). Substituting these explicit expressions into conditions (2.1) and (2.2), we may derive convergence-stable (CS) [58,59] sex-allocation strategies for high- and low-fecundity mothers (figure 1*a*,*c*; see also electronic supplementary material, figure H2).

In the context of full dispersal of females following mating (*d* = 1), we recover the CMH result: the number of sons produced by high- and low-fecundity females is exactly the same, and is independent of the difference in their fecundity (figure 1*b*,*d*; [3,5]). This can be understood by noting that the rarer-sex-effect terms (i.e. the first two terms) in inequalities (2.1) and (2.2) are exactly the same for high- and low-fecundity mothers, and that the LRC-effect terms (i.e. the final term) in inequalities (2.1) and (2.2) are both zero (because there is no LRC in the context of full dispersal of females). Accordingly, at evolutionary equilibrium (i.e. the point at which both inequalities become equations), the LMC terms (i.e. the third term) in inequalities (2.1) and (2.2) must be equal for both high- and low-fecundity mothers. Thus, *u*_Hm_*r*_S_ + *u*_Lm_*r*_M_ = *u*_Hm_*r*_M_ + *u*_Lm_*r*_S_, which implies *u*_Hm_ = *u*_Lm_, i.e. high- and low-fecundity mothers must be producing the same number of sons at evolutionary equilibrium. That is, in the full-dispersal scenario, the only asymmetry in the selection pressures acting upon sex allocation for high- and low-fecundity mothers is that the high-fecundity mothers produce sons who experience more LMC and, accordingly, they are selectively favoured to reduce their relative investment into sons until this asymmetry vanishes, at which point they are producing the same number of sons as the low-fecundity females.

However, in the context of incomplete dispersal of females following mating (*d* < 1), we find that the CMH result breaks down: high-fecundity mothers are favoured to produce more sons than are low-fecundity mothers (figure 1*b*,*d*). This difference owes to the LRC terms (i.e. the fourth term) in inequalities (2.1) and (2.2) taking non-zero values, that depend not only on the number of sons but also on the number of daughters that each mother produces. If both mothers produce the same number of sons (i.e. *u*_Hm_ = *u*_Lm_), then the high-fecundity mother must be producing more daughters than the low-fecundity mother (i.e. *u*_Hf_ > *u*_Lf_; in particular, *u*_Hf_ = *u*_Lf_ + *s*/*n*_f_). Accordingly, high-fecundity mothers experience relatively more LRC than low-fecundity mothers, such that the former are favoured to invest relatively more into sons than are the latter (i.e. *u*_Hm_ > *u*_Lm_). That is, in addition to the asymmetric selection pressures that operate in the full-dispersal scenario (such that high-fecundity mothers are favoured to invest proportionally less into sons and, accordingly, produce the same absolute number of sons as do low-fecundity mothers; i.e. the CMH result), the limited-dispersal scenario introduces an additional asymmetry (which is that the daughters of high-fecundity mothers experience LRC more strongly than do those of low-fecundity mothers), and this leads to high-fecundity mothers producing more sons than do low-fecundity mothers (i.e. breakdown of the CMH result).

Moreover, in the absence of fecundity variation between mothers (*s* = 0), we recover the dispersal-invariance result: the number of sons produced by mothers is independent of the rate of female dispersal [22,23]. Limited dispersal of females (*d* < 1) leads to an increase in LMC (figure 1*a*,*c*), which acts to promote female bias, and also an increase in LRC (electronic supplementary material, figure H1*b*,*d*), which acts to inhibit female bias, and these two effects cancel each other out [23]. The cancellation is exact under haploidy and diploidy [23], but there is a very slight decrease in investment into sons as the population becomes more viscous under haplodiploidy [60], owing to the resulting inbreeding inflating the relative value of daughters [3,55].

However, in the context of fecundity inequality (*s* > 0), we find that the dispersal-invariance result breaks down: a decrease in dispersal rate (lower *d*) leads to an increase in both LMC and LRC (electronic supplementary material, figure H1) and, although in the context of all mothers having the same fecundity (*s* = 0) these two effects cancel each other out, in the context of fecundity inequality (*s* > 0) the LRC effect is relatively stronger than the LMC effect for high-fecundity mothers, and the LRC effect is relatively weaker than the LMC effect for low-fecundity mothers (electronic supplementary material, figure H1), as explained above. In addition, although these forces are opposite in direction, they are not symmetric, and therefore the average investment into sons does not remain constant as the population becomes more viscous. Accordingly, under haploidy and diploidy, high-fecundity mothers are favoured to invest relatively more into sons in viscous populations (figure 1*b*); although, under haplodiploidy, the increased value of daughters under inbreeding means they actually invest relatively less into sons (figure 1*d*). Under haploidy, diploidy and haplodiploidy, low-fecundity mothers are favoured to invest relatively less into sons in viscous populations (figure 1*b*,*d*). Moreover, the average investment into sons across all mothers in the population decreases as the population becomes increasingly viscous (lower *d*; figure 1*b*,*d*).

### (g) Model extensions

Following the standard CMH scenario [3–5], we have assumed that each female may facultatively adjust her sex allocation according to her own fecundity status. However, for completeness, we now also consider the consequences of obligate sex allocation that is not adjusted according to a female's fecundity status. Here, the selective forces acting on the obligate sex-allocation strategy are an average of those acting on high- and low-fecundity mothers separately. Weighting the LHS's of inequalities (2.1) and (2.2) by the relative reproductive values of high- and low-fecundity mothers (i.e. 1 and 1 − *s*, respectively), and adding them together, yields a single condition for an increase in the obligate sex-allocation strategy (see electronic supplementary material, appendix, for details).

Perhaps surprisingly, although we found above that the population average of the optimal facultative investment into sons decreases as the population becomes increasingly viscous (lower *d*; figure 1)—i.e. failure of the dispersal-invariance result—we now find that the optimal obligate investment into sons is independent of the dispersal rate (*d*; electronic supplementary material, figure H3)—i.e. recovery of the dispersal-invariance result. In particular, the invariance is exact under haploidy and diploidy (electronic supplementary material, figure H3*a*,*b*), and the CS proportional investment into sons is given by z* = (1 − *s*)/(2 − *s*)^2^ (see electronic supplementary material, appendix, for details). Setting *s* = 0 recovers Hamilton's [2] z* = 1/4 result for two equally fecund mothers under full dispersal (*d* = 1), which Bulmer [22] and Frank [23] showed also extends to limited dispersal (*d* < 1), and which has been generalized here to extend to variance in maternal fecundity (*s* > 0). It also recovers Charnov's [61] result z* = (*n* − 1 − (*σ*^2^/*μ*^2^))/2*n* for full dispersal (*d* = 1) where there are *n* = 2 mothers in each patch and the coefficient of variation in fecundity is *σ*^2^/*μ*^2^ = *s*^2^/(2 − *s*)^2^, and extends this to viscous population settings (*d* < 1). There is a very slight tendency for reduced investment into sons being favoured as populations become more viscous (lower *d*) under haplodiploidy, owing to the inflation in the value of daughters under inbreeding (electronic supplementary material, figure H3*c*,*d*), and this recovers the result obtained by Taylor [60] in the absence of fecundity variation. This surprising mismatch owes to the optimal sex allocation being a nonlinear function of a mother's fecundity in viscous populations, such that the average sex allocation employed by mothers who respond facultatively to their own fecundity is distinct from the optimal sex allocation for a mother who is ignorant of (or cannot respond to information concerning) her own fecundity (note that an identical mismatch also occurs in the context of dispersal evolution [62]).

**Figure 2.**
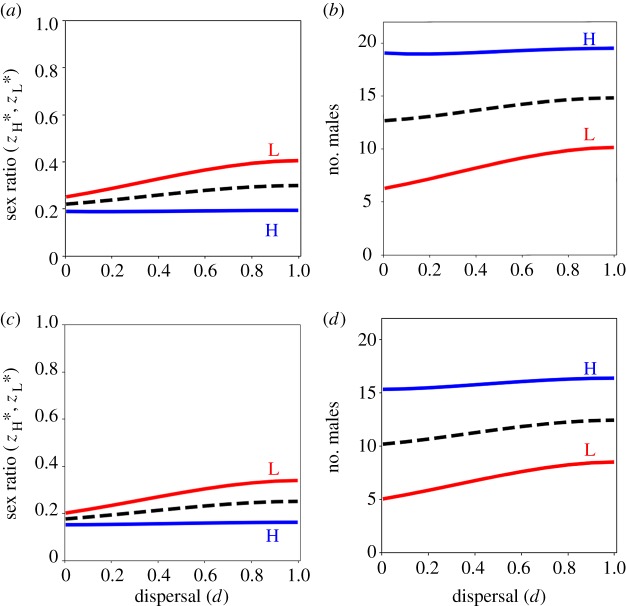
Facultative sex allocation under self-knowledge. (*a*,*c*) High-fecundity mothers (blue line, H) are favoured to produce relatively less female-biased sex ratios, whereas low-fecundity mothers (red line, L) are favoured to produce relatively more female-biased sex ratios, in viscous populations (*d* < 1), and the population average sex ratio strategy (dashed line) becomes more female-biased as the population becomes more viscous (lower *d*), under haploidy and diploidy (*a*), and under haplodiploidy (*c*). (*b*,*d*) High-fecundity mothers (blue line, H) are favoured to invest relatively more into sons than are low-fecundity mothers (red line, L) in viscous populations (*d* < 1), and the population average allocation to sons (dashed line) decreases as the population becomes more viscous (lower *d*), under haploidy and diploidy (*b*), and under haplodiploidy (*d*). We arbitrarily set the total number of offspring of a high-fecundity mother to 100. Parameter values: *k* = 0, *ρ* = 0.5, *s* = 0.75.

We have also assumed that there is one high-fecundity mother and one low-fecundity mother in every patch, such that a female's knowledge of her own fecundity immediately provides knowledge of her patchmate's fecundity. Accordingly, the facultative adjustment scenario falls within a category of models that have been termed ‘complete knowledge’ models [9]. An alternative model could consider that each female's fecundity is determined independently of her patchmates', such that some patches would contain two high-fecundity mothers, some would contain two low-fecundity mothers, and others would contain one high-fecundity mother and one low-fecundity mother. This scenario may then fall within a category of models that have been termed ‘self-knowledge’ models [9]. This could occur, for example, if mothers cannot assess the fecundity of other mothers directly, or if mothers decide their sex allocation before they encounter their patchmates [9]. If we assume that a fraction *ρ* of newborn females become high-fecundity mothers and a fraction 1 − *ρ* become low-fecundity mothers, independently of where and with whom they settle to breed, and that a focal female is aware of her own fecundity but not the fecundity of her patchmate, then we find that high-fecundity mothers are favoured to produce more sons than are low-fecundity mothers and that high-fecundity mothers are favoured to invest relatively more into sons as the population becomes more viscous (figure 2). That the CMH invariant breaks down in this scenario was already known for the special case of full dispersal (*d* = 1; [9]), and here we have also shown that the dispersal invariant breaks down in the context of variable fecundity as well.

## 3. Discussion

The enormously successful theory of sex allocation has yielded two striking invariants concerning the number of sons that a mother should produce. First, mothers are expected to produce the same number of sons, irrespective of their fecundity [3,5]. Second, mothers are expected to produce the same number of sons, irrespective of their dispersal rate [22,23,60]. Here, we have shown that these two invariance results break down when variation in maternal fecundity and limited female dispersal are considered in conjunction with each other. In particular, limited female dispersal leads to related females competing with each other for breeding opportunities, which acts to inhibit the evolution of female-biased sex allocation, and this effect becomes stronger as a mother's fecundity increases, such that high-fecundity mothers are favoured to invest relatively more into sons and low-fecundity mothers are favoured to invest relatively more into daughters, with the overall consequence that population sex ratio becomes increasingly biased towards females.

Our main focus has been on the simplest scenario of one high-quality mother and one low-quality mother breeding in every patch, with every female being able to facultatively adjust her sex allocation in response to her own fecundity. But we have also investigated the consequences of relaxing various model assumptions. First, we have investigated scenarios where mothers lack information about both their own fecundity and the fecundity of their patchmates. We have found that under this scenario we recover the result that the sex-allocation strategy does not change with population viscosity. Second, we considered scenarios where mothers know their own fecundity, but lack information about the fecundity of their patch-mates. We find that although this has a quantitative impact on the analysis, our main qualitative results are unaffected by this alteration of model assumptions. Accordingly, these results may apply to a broad range of species that differ in the details of their life cycles.

A wealth of empirical work has tested the CMH [5,10,12–21]. There is often a qualitative fit between the predictions of the theory and empirical data, and in some cases there is also a good quantitative fit (reviewed by West [1]). The results of the present analysis may help to explain those instances where the fit between theory and data is relatively poor. In this vein, we encourage empiricists to measure and report the degree of viscosity or pattern of dispersal in their study species. By contrast, theoretical and empirical work exploring the impact of population viscosity on sex allocation is relatively lacking, and we therefore also encourage more research focus on this front. Our present results caution against the idea that sex ratios are unaffected by the degree of population viscosity [22,23,60] and lend formal support to recent speculation that limited dispersal may explain the ‘scandalous’ sex ratios of *Melittobia* parasitoid wasps [63] as well as the evolution of paternal genome elimination—and concomitant sex-ratio bias—in many small arthropods [64].

More generally, the failure of a life-history invariant may both impede and facilitate the interplay of theoretical and empirical evolutionary research. The typical means of testing evolutionary theory is by use of the comparative approach [65], in which—in contrast to experimental manipulations—there may be simultaneous variation in many ecological and demographic variables. Accordingly, on the one hand, the failure of life-history invariants means that extraneous variables are more likely to be confounding, making patterns of key interest more difficult to discern; and, on the other hand, owing to the difficulties in formulating an appropriate null model, life-history invariants can be difficult to demonstrate empirically with statistical rigour [66], and so their failure—and the consequent predicted dependency of the phenotype upon a further ecological or demographic variable—provides further means of putting evolutionary theory to a proper empirical test.

Furthermore, the results of the present analysis highlight the importance of variation in individual quality in mediating not only the evolution of sex allocation, but also the evolution of other social behaviours that have fitness consequences for individuals in addition to the actor. Traditionally, applications of the comparative approach to testing social evolution theory have focused upon how differences between populations—in terms of their genetics, ecology and demography—are associated with differences in the level and types of social behaviour exhibited by those populations. However, the present analysis emphasizes the important role of differences in individual quality in mediating differences in social behaviour at the within-population level (e.g. mothers achieving different fecundities adopt different sex-allocation behaviours) and at the between-population level (e.g. populations in which there is more variation in maternal fecundity exhibit different average sex-allocation behaviours). Moreover, we have demonstrated the importance of phenotypic plasticity in driving such differences, with qualitatively different predictions emerging—concerning differences within and between populations—according to whether individuals are obliged to adopt fixed behaviours or else are able to adjust their behaviours to their particular circumstances.

## Supplementary Material

Electronic Supplementary Material

## Data accessibility

No data were used in this paper.
