# Supplementary material for: Simultaneous failure of two sex-allocation invariants: implications for sex-ratio variation within and between populations
Source: Proc Biol Sci. 2015 Jul 7;282(1810):20150570. doi: 10.1098/rspb.2015.0570 (PMC4590475; doi:10.1098/rspb.2015.0570)
Supplement: Electronic Supplementary Material [file rspb20150570supp1.docx]

**Electronic supplementary material to: “Simultaneous failure of two sex-allocation invariants”**

António M. M. Rodrigues^1,2,^*, Andy Gardner^2^

1. Department of Zoology, University of Cambridge, Downing Street, CB2 3EJ Cambridge, United Kingdom.

2. Wolfson College, Barton Road, Cambridge, CB3 9BB United Kingdom.

3. School of Biology, University of St Andrews, Dyers Brae, St Andrews KY16 9TH United Kingdom.

* Corresponding author, email: ammr3@cam.ac.uk

**Contents**

Appendix A. Reproductive success

Appendix B. Fitness

Appendix C. Stable class-frequency and reproductive value

Appendix D. Relatedness

Appendix E. Selection gradient

Appendix F. Convergence stability

Appendix G. Self-knowledge model

Appendix H. Supplementary figures (Figure H1 & H2 & H3)

References

**Appendix A. Reproductive success**

Here, we define the class-specific reproductive success of a focal breeder who allocates a proportion *x*_H_ of their reproductive resources to sons when they are high-fecundity and who allocates a proportion *x*_L_ of their reproductive resources to sons when they are of low fecundity. This depends on the quality of the focal breeder (i.e. if it is high- or low-fecundity) and on the quality of the breeder’s successful offspring (i.e. if they are either high- or low-fecundity). For convenience let us define the quantities *q*(*x*_H_,*x*_L_), and *a*(*x*_H_,*x*_L_) as

$q\left( x_{H},x_{L} \right)=\frac{1}{\left( \left( 1-x_{H} \right)+\left( 1-x_{L} \right)\left( 1-s \right) \right)\left( 1-d \right)+\left( \left( 1-z_{H} \right)+\left( 1-z_{L} \right)\left( 1-s \right) \right)d\left( 1-k \right)}$, and (A1)

$a\left( x_{H},x_{L} \right)=\left( 1-d \right)q\left( x_{H},x_{L} \right)+d\left( 1-k \right)q\left( z_{H},z_{L} \right)$, (A2)

respectively. The reproductive success of a high-fecundity female is then given by *w*_Hf🡪Hf_ = (1-*x*_H_)*a*(*x*_H_,*x*_L_)(1-*ϕ*), *w*_Hf🡪Lf_ = (1-*x*_H_)*a*(*x*_H_,*x*_L_)(1-*ϕ*), *w*_Hf🡪Hm_ = (1-*x*_H_)*a*(*x*_H_,*x*_L_)*μ*, and *w*_Hf🡪Lm_ = (1-*x*_H_)*a*(*x*_H_,*x*_L_)*μ*, through her successful offspring that become high-fecundity breeding females, low-fecundity breeding females, high-fecundity breeding males, and low-fecundity breeding males, respectively. The fraction of genes a son (or a daughter) inherits from her mother (or father) is denoted by *μ* (or *ϕ*).

The reproductive success of a low-fecundity female is given by *w*_Lf🡪Hf_ = (1-*s*)(1-*x*_L_)*a*(*x*_H_,*x*_L_)(1-*ϕ*), *w*_Lf🡪Lf_ = (1-*s*)(1-*x*_L_)*a*(*x*_H_,*x*_L_)(1-*ϕ*), *w*_Lf🡪Hm_ = (1-*s*)(1-*x*_L_)*a*(*x*_H_,*x*_L_)*μ*, and *w*_Lf🡪Lm_ = (1-*s*)(1-*x*_L_)*a*(*x*_H_,*x*_L_)*μ*, through her successful offspring that become high-fecundity breeding females, low-fecundity breeding females, high-fecundity breeding males, and low-fecundity breeding males, respectively.

The reproductive success of a high-fecundity male is given by *w*_Hm🡪Hf_ = (*x*_H_/(*x*_H_+(1-*s*)*x*_L_))(1-*x*_H_+(1-*x*_L_)(1-*s*))*a*(*x*_H_,*x*_L_)*ϕ*, *w*_Hm🡪Lf_ = (*x*_H_/(*x*_H_+(1-*s*)*x*_L_))(1-*x*_H_+(1-*x*_L_)(1-*s*))*a*(*x*_H_,*x*_L_)*ϕ*, *w*_Hm🡪Hm_ = (*x*_H_/(*x*_H_+(1-*s*)*x*_L_))(1-*x*_H_+(1-*x*_L_)(1-*s*))*a*(*x*_H_,*x*_L_)(1-*μ*), and *w*_Hm🡪Lm_ = (*x*_H_/(*x*_H_+(1-*s*)*x*_L_))(1-*x*_H_+(1-*x*_L_)(1-*s*))*a*(*x*_H_,*x*_L_)(1-*μ*), through his successful offspring that become high-fecundity breeding females, low-fecundity breeding females, high-fecundity breeding males, and low-fecundity breeding males, respectively.

The reproductive success of a low-fecundity male is given by *w*_Lm🡪Hf_ = ((1-*s*)*x*_L_/(*x*_H_+(1-*s*)*x*_L_))(1-*x*_H_+(1-*x*_L_)(1-*s*))*a*(*x*_H_,*x*_L_)*ϕ*, *w*_Lm🡪Lf_ = ((1-*s*)*x*_L_/(*x*_H_+(1-*s*)*x*_L_))(1-*x*_H_+(1-*x*_L_)(1-*s*))*a*(*x*_H_,*x*_L_)*ϕ*, *w*_Lm🡪Hm_ = ((1-*s*)*x*_L_/(*x*_H_+(1-*s*)*x*_L_))(1-*x*_H_+(1-*x*_L_)(1-*s*))*a*(*x*_H_,*x*_L_)(1-*μ*), and *w*_Lm🡪Lm_ = ((1-*s*)*x*_L_/(*x*_H_+(1-*s*)*x*_L_))(1-*x*_H_+(1-*x*_L_)(1-*s*))*a*(*x*_H_,*x*_L_)(1-*μ*), through his successful offspring that become high-fecundity breeding females, low-fecundity breeding females, high-fecundity breeding males, and low-fecundity breeding males, respectively.

The expressions of the class-specific reproductive success define a fitness matrix **A**, which is given by

$\mathbf{A}=\left( \begin{matrix} w_{Hf\to Hf} & w_{Lf\to Hf} & w_{Hm\to Hf} & w_{Lm\to Hf} \\ w_{Hf\to Lf} & w_{Lf\to Lf} & w_{Hm\to Lf} & w_{Lm\to Lf} \\ w_{Hf\to Hm} & w_{Lf\to Hm} & w_{Hm\to Hm} & w_{Lm\to Hm} \\ w_{Hf\to Lm} & w_{Lf\to Lm} & w_{Hm\to Lm} & w_{Lm\to Lm} \end{matrix} \right)$. (A3)

**Appendix B. Fitness**

Here we define the fitness of an individual according to its condition. This depends on the contribution of an individual to each class, weighted by the corresponding reproductive values, and divided by the mean reproductive value of the focal class (Taylor & Frank 1996; Frank 1998). For example, the fitness of a high-quality female is given by her contribution in terms of offspring to the different classes (as given by the expressions of reproductive success derived above), weighted by the reproductive value of these classes, divided by the mean reproductive value of a high-quality female. This is given by

$W_{\mathrm{Hf}}=\frac{v_{\mathrm{Hf}}w_{Hf\to Hf}+v_{\mathrm{Lf}}w_{Hf\to Lf}+v_{\mathrm{Hm}}w_{Hf\to Hm}+v_{\mathrm{Lm}}w_{Hf\to Lm}}{v_{\mathrm{Hf}}}$. (B1)

The fitness of a focal individual in each one of the other classes is derived in a similar way. The expected fitness of a random individual in the population is given by the class-specific fitness weighted by the frequency (*u*) and reproductive value (*v*) of each class (Taylor & Frank 1996; Frank 1998). This is

$W=u_{\mathrm{fH}}v_{\mathrm{fH}}W_{\mathrm{fH}}+u_{\mathrm{mH}}v_{\mathrm{mH}}W_{\mathrm{mH}}+u_{\mathrm{fL}}v_{\mathrm{fL}}W_{\mathrm{fL}}+u_{\mathrm{mL}}v_{\mathrm{mL}}W_{\mathrm{mL}}$. (B2)

Expanding the right hand side of this equation, we show that the expected fitness of a focal individual is given by

$W=c_{f}\left( \left( 1-x_{H} \right)k\left( x_{H},x_{L} \right)+\left( 1-x_{L} \right)\left( 1-s \right)a\left( x_{H},x_{L} \right) \right)+c_{m}\left( \frac{x_{H}}{x_{H}+x_{L}\left( 1-s \right)}\left( 1-x_{H}+\left( 1-x_{L} \right)\left( 1-s \right) \right)a\left( x_{H},x_{L} \right)+\frac{x_{L}\left( 1-s \right)}{x_{H}+x_{L}\left( 1-s \right)}\left( 1-x_{H}+\left( 1-x_{L} \right)\left( 1-s \right) \right)a\left( x_{H},x_{L} \right) \right)$, (B3)

where *c*_f_ is the class-reproductive value of females, and *c*_m_ is the class-reproductive value of males.

**Appendix C. Stable class-frequency and reproductive value**

The frequency and the individual reproductive value of each class can be derived from the matrix **A** (defined in equation A3; Taylor & Frank 1996). More specifically, the elements of the right-eigenvector corresponding to the leading eigenvalue of matrix **A** give the stable-class frequency of each class (*u*), while the elements of the left-eigenvector corresponding to the leading eigenvalue of matrix **A** give the individual reproductive value of each class (*v*). The stable-class frequencies are given by *u*_Hf_ = *u*_Lf_ = *u*_Hm_ = *u*_Lm_ = ¼. The class-reproductive values are: (1) *c*_f_ = *μ*/(*ϕ*+*μ*) for females; and (2) *c*_m_ = *ϕ*/(*ϕ*+*μ*) for males. The individual reproductive values are then given by: (1) *v*_Hf_ = *c*_f_ (1-*z*_H_) for a high-fecundity female; (2) *v*_Lf_ = *c*_f_ (1-*z*_L_)(1-*s*) for a low-fecundity female; (3) *v*_Lf_ = *c*_m_ ( *z*_H_/(*z*_H_ + *z*_L_(1-*s*))(1-*z*_H_ + (1-*z*_L_)(1-*s*)) for a high-fecundity male; and (4) *v*_Lm_ = *c*_m_ (*z*_L_(1-*s*)/(*z*_H_ + *z*_L_(1-*s*))(1-*z*_H_ + (1-*z*_L_)(1-*s*)) for a low-fecundity male.

**Appendix D. Relatedness**

Here, we define the coefficients of relatedness between interacting individuals. We assume vanishingly small genetic variation. First, we focus on haploid inheritance, then we focus on diploid inheritance, and finally we focus on haplodiploid inheritance. We first define recursion equations that describe the dynamics of the coefficients of consanguinity between successive generations, which we then solve for equilibrium. These coefficients of consanguinity enable us to derive the coefficients of relatedness between interacting individuals (Bulmer 1994).

Haploidy

We focus on the coefficient of consanguinity between a juvenile female and a juvenile male before dispersal and before mating, which is denoted by *f*. The probability that two juveniles are offspring of the high-fecundity mother is (1-*z*_H_)/(1-*z*_H_+(1-*z*_L_)(1-*s*)) times *z*_H_/(*z*_H_+*z*_L_(1-*s*)), and the probability that two juveniles are offspring of the low-fecundity mother is ((1-*z*_L_)(1-*s*))/(1-*z*_H_+(1-*z*_L_)(1-*s*)) times *z*_L_(1-*s*)/(*z*_H_+*z*_L_(1-*s*)).With probability ½ both siblings derive the same gene, otherwise with probability ½ the genes are identical with probability *f*. The probability that two juveniles are not siblings is the probability that the juvenile female is an offspring of the high-fecundity mother and the juvenile male is an offspring of the low-fecundity mother, which is given by (1-*z*_H_)/(1-*z*_H_+(1-*z*_L_)(1-*s*)) times *z*_L_(1-*s*)/(*z*_H_+*z*_L_(1-*s*)), plus the probability that the juvenile female is an offspring of the low-fecundity mother and a juvenile male is an offspring of the high-fecundity mother, which is given by ((1-*z*_L_)(1-*s*))/(1-*z*_H_+(1-*z*_L_)(1-*s*)) times *z*_H_/(*z*_H_+*z*_L_(1-*s*)). If the two juveniles are not siblings then they may have identical genes if mothers are both natives of the same patch, which occurs with probability *φ*  = (1-*d*)^2^/(1-*kd*)^2^, times the probability that they share genes in common, which is given by *f*. The recursion equation is then given by

$f'=\left( \frac{1-z_{H}}{1-z_{H}+\left( 1-z_{L} \right)(1-s)}\frac{z_{H}}{z_{H}+z_{L}(1-s)}+\frac{\left( 1-z_{L} \right)(1-s)}{1-z_{H}+\left( 1-z_{L} \right)(1-s)}\frac{z_{L}(1-s)}{z_{H}+z_{L}(1-s)} \right)\left( \frac{1}{2}+\frac{1}{2}f \right)+\left( \frac{1-z_{H}}{1-z_{H}+\left( 1-z_{L} \right)(1-s)}\frac{z_{L}(1-s)}{z_{H}+z_{L}(1-s)}+\frac{\left( 1-z_{L} \right)(1-s)}{1-z_{H}+\left( 1-z_{L} \right)(1-s)}\frac{z_{H}}{z_{H}+z_{L}(1-s)} \right)\varphi\left( \frac{1}{4}\gamma+\frac{1}{2}f+\frac{1}{4}\eta\right)$. (D1)

Let us now focus on the coefficient of consanguinity between two juvenile females, which is denoted by *γ*. The probability that two juvenile females are offspring the high-fecundity mother is ((1-*z*_H_)/(1-*z*_H_+(1-*z*_L_)(1-*s*)))^2^, and the probability that two juvenile females are offspring of the low-fecundity mother is (((1-*z*_L_)(1-*s*))/(1-*z*_H_+(1-*z*_L_)(1-*s*)))^2^. With probability ½ both siblings derive the same gene, otherwise with probability ½ the genes are identical with probability *f*. The probability that two juveniles are not siblings is the probability that the juvenile female is an offspring of the high-fecundity mother and the other juvenile female is an offspring of the low-fecundity mother, which is given by two time (1-*z*_H_)/(1-*z*_H_+(1-*z*_L_)(1-*s*)) times ((1-*z*_L_)(1-*s*))/(1-*z*_H_+(1-*z*_L_)(1-*s*)). If the two juveniles are not siblings then they may have identical genes if mothers are both natives of the same patch, which occurs with probability *φ*, times the probability that they share genes in common. With probability ¼ they both derive maternal genes, and thus the probability that they share the same gene is *γ*. With probability ½ one juvenile female derives a maternal gene, while the other derives a paternal gene, and thus the probability that they share the same gene is *f*. With probability ¼ they both derive paternal genes, and thus the probability that they share the same gene is *η*. The recursion equation is then given by

$\gamma'=\left( \left( \frac{1-z_{H}}{1-z_{H}+\left( 1-z_{L} \right)(1-s)} \right)^{2}+\left( \frac{\left( 1-z_{L} \right)(1-s)}{1-z_{H}+\left( 1-z_{L} \right)(1-s)} \right)^{2} \right)\left( \frac{1}{2}+\frac{1}{2}f \right)+\left( 2\frac{1-z_{H}}{1-z_{H}+\left( 1-z_{L} \right)(1-s)}\frac{\left( 1-z_{L} \right)(1-s)}{1-z_{H}+\left( 1-z_{L} \right)(1-s)} \right)\varphi\left( \frac{1}{4}\gamma+\frac{1}{2}f+\frac{1}{4}\eta\right)$. (D2)

Finally, let us focus on the coefficient of consanguinity between two juvenile males, which is denoted by *η*. The probability that two juvenile males are offspring of the high-fecundity mother is (*z*_H_/(*z*_H_+*z*_L_(1-*s*)))^2^, and the probability that two juvenile males are offspring of the low-fecundity mother is (*z*_L_(1-*s*)/(*z*_H_+*z*_L_(1-*s*)))^2^. With probability ½ both siblings derive the same gene, otherwise with probability ½ the genes are identical with probability *f*. The probability that two juveniles are not siblings is the probability that one is an offspring of the high-fecundity mother and the other is an offspring of the low-fecundity mother, which is given by two time *z*_H_/(*z*_H_+*z*_L_(1-*s*)) times *z*_L_(1-*s*)/(*z*_H_+*z*_L_(1-*s*)). If the two juveniles are not siblings then they may have identical genes if mothers are both natives of the same patch, which occurs with probability *φ*, times the probability that they share genes in common. With probability ¼ they both derive maternal genes, and thus the probability that they share the same gene is *γ*. With probability ½ one juvenile female derives a maternal gene, while the other derives a paternal gene, and thus the probability that they share the same gene is *f*. With probability ¼ they both derive paternal genes, and thus the probability that they share the same gene is *η*. The recursion equation is then given by

$\eta'=\left( \left( \frac{z_{H}}{z_{H}+z_{L}(1-s)} \right)^{2}+\left( \frac{z_{L}(1-s)}{z_{H}+z_{L}(1-s)} \right)^{2} \right)\left( \frac{1}{2}+\frac{1}{2}f \right)+\left( 2\frac{z_{H}}{z_{H}+z_{L}(1-s)}\frac{z_{L}(1-s)}{z_{H}+z_{L}(1-s)} \right)\varphi\left( \frac{1}{4}\gamma+\frac{1}{2}f+\frac{1}{4}\eta\right)$. (D3)

We can find the coefficient of consanguinity (*f*) by solving these recursion equations for equilibrium (i.e. by setting $f'=f$, $\gamma'=\gamma$, and $\eta'=\eta$). The coefficient of consanguinity between a mother and herself is *p* = 1*.* The coefficient of consanguinity between a mother and her daughter or son is *p*_D_ = *p*_S_ = ½*p*+ ½*f*. The coefficient of consanguinity between a mother and a daughter or son of the other mother is *p*_F_ = *p*_M_ = *φ*(½*γ*+½*f*). The relatedness between a mother and her daughters or sons is *r*_D_ = *r*_S_ = *p*_D_ / *p*. The relatedness between a mother and a daughter or son of the other mother is *r*_F_ = *r*_M_ = *p*_F_ / *p*.

Diploidy

We first focus on the coefficient of consanguinity between a juvenile female and a juvenile male before mating takes place. The probability that two juveniles are offspring of the high-fecundity mother is (1-*z*_H_)/(1-*z*_H_+(1-*z*_L_)(1-*s*)) times *z*_H_/(*z*_H_+*z*_L_(1-*s*)), and the probability that two juveniles are offspring of the low-fecundity mother is ((1-*z*_L_)(1-*s*))/(1-*z*_H_+(1-*z*_L_)(1-*s*)) times *z*_L_(1-*s*)/(*z*_H_+*z*_L_(1-*s*)).With probability ½ both siblings derive a maternal gene (or a paternal gene), in which case they are copies of the same gene with probability ½, otherwise they are identical with probability *f*. With probability ½ one sibling derives a maternal gene and the other sibling derives a paternal gene, in which case they are identical with probability *f*. The probability that two juveniles are not siblings is the probability that the juvenile female is an offspring the high-fecundity mother and the juvenile male is an offspring of the low-fecundity mother, which is given by (1-*z*_H_)/(1-*z*_H_+(1-*z*_L_)(1-*s*)) times *z*_L_(1-*s*)/(*z*_H_+*z*_L_(1-*s*)), plus the probability that the juvenile female is an offspring of the low-fecundity mother and the juvenile male is an offspring of the high-fecundity mother, which is given by ((1-*z*_L_)(1-*s*))/(1-*z*_H_+(1-*z*_L_)(1-*s*)) times *z*_H_/(*z*_H_+*z*_L_(1-*s*)). If the two juveniles are not siblings then they may have identical genes if mothers are both natives of same the patch, which occurs with probability *φ*. If both mothers are natives, then: with probability ¼ both genes are maternally derived, in which case they are identical with probability *γ*; with probability ½ one gene in maternally derived while the other is paternally derived, in which case they are identical with probability *f*; and finally with probability ¼ both genes are paternally derived, in which case they are identical with probability *η*. The recursion equation is then given by

$f'=\left( \frac{1-z_{H}}{1-z_{H}+\left( 1-z_{L} \right)\left( 1-s \right)}\frac{z_{H}}{z_{H}+z_{L}\left( 1-s \right)}+\frac{\left( 1-z_{L} \right)\left( 1-s \right)}{1-z_{H}+\left( 1-z_{L} \right)\left( 1-s \right)}\frac{z_{L}\left( 1-s \right)}{z_{H}+z_{L}\left( 1-s \right)} \right)\left( \frac{1}{2}\left( \frac{1}{2}+\frac{1}{2}f \right)+\frac{1}{2}f \right)+\left( \frac{1-z_{H}}{1-z_{H}+\left( 1-z_{L} \right)\left( 1-s \right)}\frac{z_{L}\left( 1-s \right)}{z_{H}+z_{L}\left( 1-s \right)}+\frac{\left( 1-z_{L} \right)\left( 1-s \right)}{1-z_{H}+\left( 1-z_{L} \right)\left( 1-s \right)}\frac{z_{H}}{z_{H}+z_{L}\left( 1-s \right)} \right)\varphi\left( \frac{1}{4}\gamma+\frac{1}{2}f+\frac{1}{4}\eta\right)$. (D4)

Let us now focus on the coefficient of consanguinity between two juvenile females. The probability that two juvenile females are offspring of the high-fecundity mother is ((1-*z*_H_)/(1-*z*_H_+(1-*z*_L_)(1-*s*)))^2^, and the probability that two juvenile females are offspring of the low-fecundity mother is (((1-*z*_L_)(1-*s*))/(1-*z*_H_+(1-*z*_L_)(1-*s*)))^2^. With probability ¼ they both derive a maternal gene, in which case they are copies of the same gene with probability ½, otherwise they are identical with probability *f*. With probability ½ one the juvenile female derives a paternal gene, while the other derives a maternal gene, in which case they are identical with probability *f*. With probability ¼ they both derive a paternal gene, in which case they are copies of the same gene with probability ½, otherwise they are identical with probability *f*. The probability that two juveniles are not siblings is the probability that one juvenile female is an offspring of the high-fecundity mother and the other juvenile female is an offspring of the low-fecundity mother, which is given by two time (1-*z*_H_)/(1-*z*_H_+(1-*z*_L_)(1-*s*)) times ((1-*z*_L_)(1-*s*))/(1-*z*_H_+(1-*z*_L_)(1-*s*)). If the two juveniles are not siblings then they may have identical genes if mothers are both natives of the same patch, which occurs with probability *φ*, times the probability that they share genes in common. With probability ¼ they both derive maternal genes, and thus the probability that they share the same gene is *γ*. With probability ½ one juvenile female derives a maternal gene, while the other derives a paternal gene, and thus the probability that they share the same gene is *f*. With probability ¼ they both derive paternal genes, and thus the probability that they share the same gene is *η*. The recursion equation is then given by

$\gamma'=\left( \left( \frac{1-z_{H}}{1-z_{H}+\left( 1-z_{L} \right)(1-s)} \right)^{2}+\left( \frac{\left( 1-z_{L} \right)(1-s)}{1-z_{H}+\left( 1-z_{L} \right)(1-s)} \right)^{2} \right)\left( \frac{1}{2}\left( \frac{1}{2}+\frac{1}{2}f \right)+\frac{1}{2}f \right)+\left( 2\frac{1-z_{H}}{1-z_{H}+\left( 1-z_{L} \right)(1-s)}\frac{\left( 1-z_{L} \right)(1-s)}{1-z_{H}+\left( 1-z_{L} \right)(1-s)} \right)\varphi\left( \frac{1}{4}\gamma+\frac{1}{2}f+\frac{1}{4}\eta\right)$. (D5)

Finally, let us focus on the coefficient of consanguinity between two juvenile males. The probability that two juvenile males are offspring of the high-fecundity mother is (*z*_H_/(*z*_H_+*z*_L_(1-*s*)))^2^, and the probability that two juvenile males are offspring of the low-fecundity mother is (*z*_L_(1-*s*)/(*z*_H_+*z*_L_(1-*s*)))^2^. With probability ¼ they both derive a maternal gene, in which case they are copies of the same gene with probability ½, otherwise they are identical with probability *f*. With probability ½ one the juvenile female derives a paternal gene, while the other derives a maternal gene, in which case they are identical with probability *f*. With probability ¼ they both derive a paternal gene, in which case they are copies of the same gene with probability ½, otherwise they are identical with probability *f*. The probability that two juveniles are not siblings is the probability that one is an offspring of the high-fecundity mother and the other is an offspring of the low-fecundity mother, which is given by two time *z*_H_/(*z*_H_+*z*_L_(1-*s*)) times *z*_L_(1-*s*)/(*z*_H_+*z*_L_(1-*s*)). If the two juveniles are not siblings then they may have identical genes if mothers are both natives of the same patch, which occurs with probability *φ*, times the probability that they share genes in common. With probability ¼ they both derive maternal genes, and thus the probability that they share the same gene is *γ*. With probability ½ one juvenile female derives a maternal gene, while the other derives a paternal gene, and thus the probability that they share the same gene is *f*. With probability ¼ they both derive paternal genes, and thus the probability that they share the same gene is *η*. The recursion equation is then given by

$\eta'=\left( \left( \frac{z_{H}}{z_{H}+z_{L}(1-s)} \right)^{2}+\left( \frac{z_{L}(1-s)}{z_{H}+z_{L}(1-s)} \right)^{2} \right)\left( \frac{1}{2}\left( \frac{1}{2}+\frac{1}{2}f \right)+\frac{1}{2}f \right)+\left( 2\frac{z_{H}}{z_{H}+z_{L}(1-s)}\frac{z_{L}(1-s)}{z_{H}+z_{L}(1-s)} \right)\varphi\left( \frac{1}{4}\gamma+\frac{1}{2}f\frac{1}{4}\eta\right)$. (D6)

We can find the coefficient of consanguinity *f* by solving these recursion equations for equilibrium (i.e. by setting $f'=f$, $\gamma'=\gamma$, and $\eta'=\eta$). The coefficient of consanguinity between a mother and herself is *p* = ½ + ½*f.* The coefficient of consanguinity between a mother and her daughter or son is *p*_D_ = *p*_S_ = ½*p*+ ½*f*. The coefficient of consanguinity between a mother and a daughter or son of the other mother is *p*_F_ = *p*_M_ = *φ*(½*γ*+½*f*). The relatedness between a mother and her daughters or sons is *r*_D_ = *r*_S_ = *p*_D_ / *p*. The relatedness between a mother and a daughter or son of the other mother is *r*_F_ = *r*_M_ = *p*_F_ / *p*.

Haplodiploidy

We first focus on the coefficient of consanguinity between two mating partners, this is the coefficient of consanguinity between opposite-sex juveniles in a focal patch before dispersal. The probability that two opposite-sex juveniles are offspring of to the high-fecundity mother is (1-*z*_H_)/(1-*z*_H_+(1-*z*_L_)(1-*s*)) times *z*_H_/(*z*_H_+*z*_L_(1-*s*)), and the probability that two juveniles are offspring of the low-fecundity mother is ((1-*z*_L_)(1-*s*))/(1-*z*_H_+(1-*z*_L_)(1-*s*)) times *z*_L_(1-*s*)/(*z*_H_+*z*_L_(1-*s*)). The juvenile male derives his gene from the mother. With probability ½ juvenile female also derives a maternal gene, in which case they are copies of the same gene with probability ½, otherwise they are identical with probability *f*. With probability ½ one the juvenile female derives a paternal gene, in which case they are identical with probability *f*. The probability that two juveniles are not siblings is the probability that the juvenile female is an offspring of the high-fecundity mother and the juvenile male is an offspring of the low-fecundity mother, which is given by (1-*z*_H_)/(1-*z*_H_+(1-*z*_L_)(1-*s*)) times *z*_L_(1-*s*)/(*z*_H_+*z*_L_(1-*s*)), plus the probability that the juvenile female is offspring of the low-fecundity mother and the juvenile male is an offspring of the high-fecundity mother, which is given by ((1-*z*_L_)(1-*s*))/(1-*z*_H_+(1-*z*_L_)(1-*s*)) times *z*_H_/(*z*_H_+*z*_L_(1-*s*)). If the two juveniles are not siblings then they may have identical genes if mothers are both natives of the same patch, which occurs with probability *φ*, times the probability that they share genes in common. With probability ½ the juvenile female gene is maternally derived, and thus the probability that she has the same gene than the juvenile male is *γ*. With probability ½ the juvenile female gene is paternally derived, and thus the probability that she has the same gene than the juvenile male is *f*. The recursion equation is then given by

$f'=\left( \frac{1-z_{H}}{1-z_{H}+\left( 1-z_{L} \right)(1-s)}\frac{z_{H}}{z_{H}+z_{L}(1-s)}+\frac{\left( 1-z_{L} \right)(1-s)}{1-z_{H}+\left( 1-z_{L} \right)(1-s)}\frac{z_{L}(1-s)}{z_{H}+z_{L}(1-s)} \right)\left( \frac{1}{2}\left( \frac{1}{2}+\frac{1}{2}f \right)+\frac{1}{2}f \right)+\left( \frac{1-z_{H}}{1-z_{H}+\left( 1-z_{L} \right)(1-s)}\frac{z_{L}(1-s)}{z_{H}+z_{L}(1-s)}+\frac{\left( 1-z_{L} \right)(1-s)}{1-z_{H}+\left( 1-z_{L} \right)(1-s)}\frac{z_{H}}{z_{H}+z_{L}(1-s)} \right)\varphi\left( \frac{1}{2}\gamma+\frac{1}{2}f \right)$. (D7)

Let us now focus on the coefficient of consanguinity between two juvenile females. The probability that two juvenile females are offspring of the high-fecundity mother is ((1-*z*_H_)/(1-*z*_H_+(1-*z*_L_)(1-*s*)))^2^, and the probability that two juvenile females are offspring of the low-fecundity mother is (((1-*z*_L_)(1-*s*))/(1-*z*_H_+(1-*z*_L_)(1-*s*)))^2^. With probability ¼ they both derive a maternal gene, in which case they are copies of the same gene with probability ½, otherwise they are identical with probability *f*. With probability ½ one the juvenile female derives a paternal gene, while the other derives a maternal gene, in which case they are identical with probability *f*. With probability ¼ they both derive a paternal gene, in which case they are copies of the same gene. The probability that two juveniles are not siblings is the probability that one juvenile female is offspring of the high-fecundity mother and the other juvenile female is offspring of the low-fecundity mother, which is given by two time (1-*z*_H_)/(1-*z*_H_+(1-*z*_L_)(1-*s*)) times ((1-*z*_L_)(1-*s*))/(1-*z*_H_+(1-*z*_L_)(1-*s*)). If the two juveniles are not siblings then they may have identical genes if mothers are both natives to the same patch, which occurs with probability *φ*, times the probability that they share genes in common. With probability ¼ they both derive maternal genes, and thus the probability that they share the same gene is *γ*. With probability ½ one juvenile female derives a maternal gene, while the other derives a paternal gene, and thus the probability that they share the same gene is *f*. With probability ¼ they both derive paternal genes, and thus the probability that they share the same gene is *η*. The recursion equation is then given by

$\gamma'=\left( \left( \frac{1-z_{H}}{1-z_{H}+\left( 1-z_{L} \right)(1-s)} \right)^{2}+\left( \frac{\left( 1-z_{L} \right)(1-s)}{1-z_{H}+\left( 1-z_{L} \right)(1-s)} \right)^{2} \right)\left( \frac{1}{4}\left( \frac{1}{2}+\frac{1}{2}f \right)+\frac{1}{2}f+\frac{1}{4} \right)+\left( 2\frac{1-z_{H}}{1-z_{H}+\left( 1-z_{L} \right)(1-s)}\frac{\left( 1-z_{L} \right)(1-s)}{1-z_{H}+\left( 1-z_{L} \right)(1-s)} \right)\varphi\left( \frac{1}{4}\gamma+\frac{1}{2}f+\frac{1}{4}\eta\right)$. (D8)

Finally, let us focus on the coefficient of consanguinity between two juvenile males. The probability that two juvenile males are offspring of the high-fecundity mother is (*z*_H_/(*z*_H_+*z*_L_(1-*s*)))^2^, and the probability that two juvenile males are offspring of the low-fecundity mother is (*z*_L_(1-*s*)/(*z*_H_+*z*_L_(1-*s*)))^2^. With probability ½ they both derive copies of the same gene with probability ½, otherwise with probability ½ they are identical with probability *f*. The probability that two juveniles are not siblings is the probability that one is an offspring of the high-fecundity mother and the other is an offspring of the low-fecundity mother, which is given by two time *z*_H_/(*z*_H_+*z*_L_(1-*s*)) times *z*_L_(1-*s*)/(*z*_H_+*z*_L_(1-*s*)). If the two juveniles are not siblings then they may have identical genes if mothers are both natives of the same patch, which occurs with probability *φ*, times the probability that they share genes in common, which is given by *γ*. The recursion equation is then given by

$\eta'=\left( \left( \frac{z_{H}}{z_{H}+z_{L}(1-s)} \right)^{2}+\left( \frac{z_{L}(1-s)}{z_{H}+z_{L}(1-s)} \right)^{2} \right)\left( \frac{1}{2}+\frac{1}{2}f \right)+\left( 2\frac{z_{H}}{z_{H}+z_{L}(1-s)}\frac{z_{L}(1-s)}{z_{H}+z_{L}(1-s)} \right)\varphi\gamma$. (D9)

We can find these three coefficients of consanguinity by solving these three recursion equations for equilibrium (i.e. by setting $f'=f$, $\gamma'=\gamma$, and $\eta'=\eta$). The coefficient of consanguinity between a mother and herself is *p* = ½ + ½*f.* The coefficient of consanguinity between a mother and her daughter is *p*_D_ = ½*p*+ ½*f*. The coefficient of consanguinity between a mother and her son is *p*_S_ = ½ + ½*f*. The coefficient of consanguinity between a mother and the daughter of the other mother is *p*_F_ = *φ*(½*γ* + ½*f* ). The coefficient of consanguinity between a mother and the son of the other mother is *p*_M_ = *φγ*. The relatedness between a mother and her daughters is *r*_D_ = *p*_D_ / *p*, and the relatedness between a mother and her sons is *r*_S_ = *p*_S_ / *p*. The relatedness between a mother and a daughter the other mother is *r*_F_ = *p*_F_ / *p*, the relatedness between a mother and a son the other mother is *r*_M_ = *p*_M_ / *p*.

**Appendix E. Selection gradient**

The selection gradient for the sex ratio expressed conditionally on the mother’s fecundity is given by the slope of her fitness *W* on her breeding value for the sex ratio (Taylor & Frank 1996; Frank 1998). The breeding value of a high-fecundity mother is denoted by *g*_fH_, while the breeding value of a low-fecundity mother is denoted by *g*_fL_. The selection gradients are given by

$\frac{dW}{dg_{\mathrm{fH}}}=c_{f}\left( \frac{\partial}{\partial x_{H}}\left( \left( 1-x_{H} \right)a\left( x_{H},x_{L} \right) \right)r_{\mathrm{MD}}+\frac{\partial}{\partial x_{H}}\left( \left( 1-x_{L} \right)\left( 1-s \right)a\left( x_{H},x_{L} \right) \right)r_{\mathrm{MF}} \right)+c_{m}\left( \frac{\partial}{\partial x_{H}}\left( \frac{x_{H}}{x_{H}+x_{L}\left( 1-s \right)}\left( 1-x_{H}+\left( 1-x_{L} \right)\left( 1-s \right) \right)a\left( x_{H},x_{L} \right) \right)r_{\mathrm{MS}}+\frac{\partial}{\partial x_{H}}\left( \frac{x_{L}\left( 1-s \right)}{x_{H}+x_{L}\left( 1-s \right)}\left( 1-x_{H}+\left( 1-x_{L} \right)\left( 1-s \right) \right)a\left( x_{H},x_{L} \right) \right)r_{\mathrm{MM}} \right)$, and (E1)

$\frac{dW}{dg_{\mathrm{fL}}}=c_{f}\left( \frac{\partial}{\partial x_{L}}\left( \left( 1-x_{H} \right)a\left( x_{H},x_{L} \right) \right)r_{\mathrm{MF}}+\frac{\partial}{\partial x_{L}}\left( \left( 1-x_{L} \right)\left( 1-s \right)a\left( x_{H},x_{L} \right) \right)r_{\mathrm{MD}} \right)+c_{m}\left( \frac{\partial}{\partial x_{L}}\left( \frac{x_{H}}{x_{H}+x_{L}\left( 1-s \right)}\left( 1-x_{H}+\left( 1-x_{L} \right)\left( 1-s \right) \right)a\left( x_{H},x_{L} \right) \right)r_{\mathrm{MM}}+\frac{\partial}{\partial x_{L}}\left( \frac{x_{L}\left( 1-s \right)}{x_{H}+x_{L}\left( 1-s \right)}\left( 1-x_{H}+\left( 1-x_{L} \right)\left( 1-s \right) \right)a\left( x_{H},x_{L} \right) \right)r_{\mathrm{MS}} \right)$, (E2)

for a high-quality mother, and for a low-quality mother, respectively. If we expand the right-hand side of these equations, we get the left-hand side (LHS) of inequalities (1) and (2) in the main text, which are the conditions for natural selection to favour an increase in the sex allocation strategy. To determine the optimal sex allocation strategy, we set the LHS of inequalities (1) and (2) to zero, and we solve the system of equations for equilibrium.

**Appendix F. Convergence stability**

Here we determine the convergence stability (CS; Christiansen 1991; Eshel 1996; Taylor 1996) of the optimal sex allocation strategies. To determine if a pair of optimal sex allocation strategies is convergence stable we define the matrix:

$\left. \left( \begin{matrix} \frac{\partial}{\partial z_{L}}\left( \left. \frac{\partial W}{\partial g_{\mathrm{fL}}} \right|_{x_{L}=z_{L}} \right) & \frac{\partial}{\partial z_{H}}\left( \left. \frac{\partial W}{\partial g_{\mathrm{fL}}} \right|_{x_{L}=z_{L}} \right) \\ \frac{\partial}{\partial z_{L}}\left( \left. \frac{\partial W}{\partial g_{\mathrm{fH}}} \right|_{x_{H}=z_{H}} \right) & \frac{\partial}{\partial z_{H}}\left( \left. \frac{\partial W}{\partial g_{\mathrm{fH}}} \right|_{x_{H}=z_{H}} \right) \end{matrix} \right) \right|_{z_{H}=z_{H}^{*},z_{L}=z_{L}^{*}}$. (F1)

The pair of optimal strategies (*z*_H_^*^ and *z*_L_^*^) are convergence stable if both eigenvalues of matrix (F1) have negative real parts (Otto and Day 2007). If mothers are obliged to invest a fixed amount into sons, irrespective of their fecundity, then the condition for convergence stability is

$\left. \frac{\partial}{\partial z}\left( \left. \frac{\partial W}{\partial g_{f}} \right|_{z_{H}=z_{L}=z} \right) \right|_{z=z^{*}}<0$, (F2)

where *g*_f_ is the breeding value of a random a random mother in the population. We find that both the facultative and the obligate sex allocation strategies are convergence stable.

**Appendix G. Self-knowledge model**

Life-cycle and fitness

In the main text we outlined a model where all patches have one high-fecundity and one low-fecundity mother. Here we extend this model and instead of considering that all patches have one high-fecundity and one low-fecundity mother, we consider a model where the quality of each female is defined before the breeding season. Specifically, we assume that juvenile females become high-fecundity mothers with probability *ρ* and become low-fecundity mothers with probability 1-*ρ*. This means that: (1) the frequency of patches with two high-fecundity mothers is *u*_0_ = *ρ*^2^; (2) the frequency of patches with one high-fecundity mother and one low-fecundity mother is *u*_1_ = 2*ρ*(1-*ρ*); and (3) the frequency of patches with two low-fecundity mothers is *u*_2_ = (1-*ρ*)^2^. This also means that: (1) the frequency of high-fecundity mothers in patches with two high-fecundity mothers is *u*_H0_ = *ρ*^2^; the frequency of high-fecundity mothers in patches with one high-fecundity mother and one low-fecundity mother is *u*_H1_ = *ρ*(1-*ρ*); (3) the frequency of low-fecundity mothers in patches with one high-fecundity mother and one low-fecundity mother is *u*_L1_ = *ρ*(1-*ρ*); and (4) the frequency of low-fecundity mothers in patches with two low-fecundity mothers is *u*_L2_ = (1-*ρ*)^2^. Note that we now use two indices. First, we denote the quality of the mother by the letters ‘H’ (high-fecundity) and ‘L’ (low-fecundity). Second, we denote the condition of the patch by the numbers ‘0’ (patches with two high-fecundity mothers), ‘1’ (mixed patches with one high-fecundity mother and one low-fecundity mother), and ‘2’ (patches with two low-fecundity mothers).

To analyse this extended model we follow the steps delineated above. We start by defining the class-specific reproductive success of a focal breeder. This depends on the quality of the focal breeder and on the condition of the patch (i.e. ‘H’ or ‘L’, and ‘0’, ‘1’ or ‘2’) and on the quality and condition of the breeder’s successful offspring (i.e. ‘H’ or ‘L’, and ‘0’, ‘1’ or ‘2’). For convenience let us define the following quantities: 1-$\bar{z}$ = *u*_0_2(1-*z*_H0_)+*u*_1_(1-*z*_H1_+(1-*z*_L1_)(1-*s*))+ *u*_2_2(1-*z*_L2_)(1-*s*); *q*_0_(*x*_H0_,*y*_H0_) = 1/(((1-*x*_H0_)+(1-*y*_H0_))(1-*d*)+(1-$\bar{z}$)*d*(1-*k*)); *q*_1_(*x*_H1_,*x*_L1_) = 1/(((1-*x*_H1_)+(1-*x*_H1_)(1-*s*))(1-*d*)+(1-$\bar{z}$)*d*(1-*k*)); *q*_2_(*x*_L2_,*y*_L2_) = 1/(((1-*x*_L2_)+(1-*y*_L2_))(1-*s*)(1-*d*)+(1-$\bar{z}$)*d*(1-*k*)); $\bar{q}$ = *u*_0_*q*_0_(*x*_H0_,*y*_H0_)+*u*_1_*q*_1_(*x*_H1_,*x*_L1_) *u*_2_*q*_2_(*x*_L2_,*y*_L2_); *a*_0_(*x*_H0_,*y*_H0_) = (1-*d*)*q*_0_(*x*_H0_,*y*_H0_)+*d*(1-*k*)$\bar{q}$; *a*_1_(*x*_H1_,*x*_L1_) = (1-*d*)*q*_1_(*x*_H1_,*x*_L1_)+*d*(1-*k*)$\bar{q}$; *a*_2_(*x*_L2_,*y*_L2_) = (1-*d*)*q*_2_(*x*_L2_,*y*_L2_)*+d*(1-*k*)$\bar{q}$; where *x* denotes the sex ratio strategy of the focal individual, *y* denotes the sex ratio strategy of the group mate, and *z* denotes the average sex ratio strategy across the population. We can now define the fitness success of each female.

The fitness of a focal individual depends on its condition. We follow the method outlined in appendix B. The fitness of a individual, according to its condition, is given by: *W*_H0f_ = *c*_f_(1-*x*_H0_)*a*_0_(*x*_H0_,*y*_H0_)/*v*_H0f_, *W*_H1f_ = *c*_f_(1-*x*_H1_)*a*_1_(*x*_H1_,*x*_L1_)/*v*_H1f_, *W*_L1f_ = *c*_f_(1-*x*_L1_)(1-*s*)*a*_1_(*x*_H1_,*x*_L1_)/*v*_L1f_, *W*_L2f_ = *c*_f_(1-*x*_L2_)(1-*s*)*a*_2_(*x*_L2_,*y*_L2_)/*v*_L2f_, *W*_H0m_ = *c*_m_(*x*_H0_/(*x*_H0_+*y*_H0_))(1-*x*_H0_+1-*y*_H0_)*a*_0_(*x*_H0_,*y*_H0_)/*v*_H0f_, *W*_H1m_ = *c*_m_(*x*_H1_/(*x*_H1_+*x*_L1_(1-*s*)))(1-*x*_H1_+(1-*x*_L1_)(1-*s*))*a*_1_(*x*_H1_,*x*_L1_)/*v*_H1f_, *W*_L1m_ = *c*_m_(*x*_L1_(1-*s*)/(*x*_H1_+*x*_L1_(1-*s*)))(1-*x*_H1_+(1-*x*_L1_)(1-*s*))*a*_1_(*x*_H1_,*x*_L1_)/*v*_L1f_, *W*_L2m_ = *c*_m_(*x*_L2_/(*x*_L2_+*y*_L2_))(1-*x*_L2_+1-*y*_L2_)(1-*s*)*a*_2_(*x*_L2_,*y*_L2_)/*v*_L2f_.

Relatedness

We follow the approach outlined above to determine the coefficients of relatedness among interacting individuals. We focus on the coefficient of consanguinity between two opposite-sex juveniles in patches with two high-fecundity mothers (denoted by *f*_0_), in mixed patches (denoted by *f*_1_), and in patches with two low-fecundity mothers (denoted by *f*_2_); on the coefficient of consanguinity between two juvenile females in patches with two high-fecundity mothers (denoted by *γ*_0_), in mixed patches (denoted by *γ*_1_), and in patches with two low-fecundity mothers (denoted by *γ*_2_); and on the coefficient of consanguinity between two juvenile males in patches with two high-fecundity mothers (denoted by *η*_0_), in mixed patches (denoted by *η*_1_), and in patches with two low-fecundity mothers (denoted by *η*_2_). As above, we define a recursion equation for each one of these coefficients of consanguinity, and this gives us a system of equations for each type of inheritance that we then solve for equilibrium.

*Haploidy --* Here we focus on haploid inheritance. In patches with two high-fecundity females and in patches with two low-fecundity females the probability that two juveniles sampled at random are siblings is *P*_f0_ = ½. In mixed patches: (1) the probability that two juveniles of the opposite sex are siblings is given by *P*_f1_ = ((1-*z*_H1_)/(1-*z*_H1_+(1-*z*_L1_)(1-*s*)))(*z*_H1_/( *z*_H1_+*z*_L1_(1-*s*)))+( (1-*z*_L1_)(1-*s*))/(1-*z*_H1_+(1-*z*_L1_)(1-*s*)))( *z*_L1_(1-*s*)/( *z*_H1_+*z*_L1_(1-*s*))); (2) the probability that two female juveniles are siblings is given by *P*_γ1_ = ((1-*z*_H1_)/(1-*z*_H1_+(1-*z*_L1_)(1-*s*)))^2^+((1-*z*_L1_)(1-*s*))/(1-*z*_H1_+(1-*z*_L1_)(1-*s*)))^2^; and (3) the probability that two male juveniles are siblings is given by *P*_η1_ = (*z*_H1_/( *z*_H1_+*z*_L1_(1-*s*)))^2^+( *z*_H1_+*z*_L1_(1-*s*)))^2^. The probability that a female chosen at random after dispersal is native to the patch is given by: (1) *h*_0_ = 2(1-*z*_H0_)(1-*d*)*q*_0_(*z*_H0_,*z*_H0_) in patches with two high-fecundity mothers; *h*_1_ = ((1-*z*_H1_)+(1-*z*_L1_)(1-*s*))(1-*d*)*q*_1_(*z*_H1_,*z*_L1_) in mixed patches; and (3) *h*_2_ = 2(1-*z*_L2_)(1-*d*)*q*_2_(*z*_L2_,*z*_L2_) in patches with two low-fecundity mothers. Thus, the probabilities of co-philopatry are given by *φ*_0_ = *h*_0_^2^, *φ*_1_ = *h*_1_^2^, and *φ*_2_ = *h*_2_^2^. The probability that a focal patch had two high-fecundity females in the previous generation is *π*_0_ = *u*_0_. The probability that a focal patch had a high-fecundity female and a low-fecundity female is *π*_1_ = *u*_1_. The probability that a focal patch had two low-fecundity females in the previous generation is *π*_2_ = *u*_2_. The probability that a disperser was born in a patch with two high-fecundity females is *α*_0_ = (*u*_0_2(1-*z*_H0_))/(1-$\bar{z}$). The probability that a disperser was born in a patch with one high-fecundity female and one low-fecundity female is *α*_1_ = (*u*_1_((1-*z*_H1_)+(1-*z*_L1_)(1-*s*)))/(1-$\bar{z}$). The probability that a disperser was born in a patch with two low-fecundity females is *α*_2_ = (*u*_2_2(1-*z*_L2_))/(1-$\bar{z}$). The recursion equations are given by

$f_{0}'=P_{f0}\left( \frac{1}{2}+\frac{1}{2}\iota\right)+\left( 1-P_{f0} \right)\left( \left( \pi_{0}\varphi_{0}+\pi_{2}\varphi_{2} \right)f_{0}+\pi_{1}\varphi_{1}\left( \frac{1}{4}\gamma_{1}+\frac{1}{2}f_{1}+\frac{1}{4}\eta_{1} \right) \right)$, (G1)

$f_{1}'=P_{f1}\left( \frac{1}{2}+\frac{1}{2}\iota\right)+\left( 1-P_{f1} \right)\left( \left( \pi_{0}\varphi_{0}+\pi_{2}\varphi_{2} \right)f_{0}+\pi_{1}\varphi_{1}\left( \frac{1}{4}\gamma_{1}+\frac{1}{2}f_{1}+\frac{1}{4}\eta_{1} \right) \right)$, (G2)

$\gamma_{1}'=P_{\gamma1}\left( \frac{1}{2}+\frac{1}{2}\iota\right)+\left( 1-P_{\gamma1} \right)\left( \left( \pi_{0}\varphi_{0}+\pi_{2}\varphi_{2} \right)f_{0}+\pi_{1}\varphi_{1}\left( \frac{1}{4}\gamma_{1}+\frac{1}{2}f_{1}+\frac{1}{4}\eta_{1} \right) \right)$, and (G3)

$\eta_{1}'=P_{\eta1}\left( \frac{1}{2}+\frac{1}{2}\iota\right)+\left( 1-P_{\eta1} \right)\left( \left( \pi_{0}\varphi_{0}+\pi_{2}\varphi_{2} \right)f_{0}+\pi_{1}\varphi_{1}\left( \frac{1}{4}\gamma_{1}+\frac{1}{2}f_{1}+\frac{1}{4}\eta_{1} \right) \right)$,(G4)

in which the coefficient of inbreeding (denoted by *ι*) is given by

$\iota=\left( \pi_{0}\left( 1-h_{0} \right)+\pi_{1}\left( 1-h_{1} \right)+\pi_{2}\left( 1-h_{2} \right) \right)\left( \alpha_{0}f_{0}+\alpha_{1}f_{1}+\alpha_{2}f_{2} \right)+\pi_{0}h_{0}f_{0}+\pi_{1}h_{1}f_{1}+\pi_{2}h_{2}f_{0}$. (G5)

Note that we only need four recursion equations, as the recursion equations for the coefficients of consanguinity *γ*_0_, *η*_0_*, f*_2_, *γ*_2_, and *η*_2_ are all identical to the recursion equation for the coefficient of consanguinity *f*_0_. We can find the coefficients of consanguinity by solving these recursion equations for equilibrium. The coefficient of consanguinity between a mother and herself is *p* = 1*.* The coefficient of consanguinity between a mother and her daughter or son is *p*_D_ = *p*_S_ = ½*p*_M_+ ½*ι*. The coefficient of consanguinity between a mother and a daughter or son of the other mother is *p*_F_ = *p*_M_ = (*π*_0_*φ*_0_+ *π*_2_*φ*_2_)*f*_0_+*π*_1_*φ*_1_(½*γ*_1_+½*f*_1_). The relatedness between a mother and her daughters or sons is *r*_D_ = *r*_S_ = *p*_D_ / *p*. The relatedness between a mother and a daughter or son of the other mother is *r*_F_ = *r*_M_ = *p*_F_ / *p*.

*Diploidy* -- Here we focus on diploid inheritance. In patches with two high-fecundity females and in patches with two low-fecundity females the probability two juveniles sampled at random are siblings is *P*_f0_ = ½. In mixed patches: (1) the probability that two juveniles of the opposite sex are siblings is given by *P*_f1_ = ((1-*z*_H1_)/(1-*z*_H1_+(1-*z*_L1_)(1-*s*)))(*z*_H1_/( *z*_H1_+*z*_L1_(1-*s*)))+( (1-*z*_L1_)(1-*s*))/(1-*z*_H1_+(1-*z*_L1_)(1-*s*)))( *z*_L1_(1-*s*)/( *z*_H1_+*z*_L1_(1-*s*))); (2) the probability that two female juveniles are siblings is given by *P*_γ1_ = ((1-*z*_H1_)/(1-*z*_H1_+(1-*z*_L1_)(1-*s*)))^2^+((1-*z*_L1_)(1-*s*))/(1-*z*_H1_+(1-*z*_L1_)(1-*s*)))^2^; and (3) the probability that two male juveniles are siblings is given by *P*_η1_ = (*z*_H1_/( *z*_H1_+*z*_L1_(1-*s*)))^2^+( *z*_H1_+*z*_L1_(1-*s*)))^2^. The probability that a female chosen at random after dispersal is native to the patch is given by: (1) *h*_0_ = 2(1-*z*_H0_)(1-*d*)*q*_0_(*z*_H0_,*z*_H0_) in patches with two high-fecundity mothers; *h*_1_ = ((1-*z*_H1_)+(1-*z*_L1_)(1-*s*))(1-*d*)*q*_1_(*z*_H1_,*z*_L1_) in mixed patches; and (3) *h*_2_ = 2(1-*z*_L2_)(1-*d*)*q*_2_(*z*_L2_,*z*_L2_) in patches with two low-fecundity mothers. Thus, the probabilities of co-philopatry are given by *φ*_0_ = *h*_0_^2^, *φ*_1_ = *h*_1_^2^, and *φ*_2_ = *h*_2_^2^. The probability that a focal patch had two high-fecundity females in the previous generation is *π*_0_ = *u*_0_. The probability that a focal patch had a high-fecundity female and a low-fecundity female is *π*_1_ = *u*_1_. The probability that a focal patch had two low-fecundity females in the previous generation is *π*_2_ = *u*_2_. The probability that a disperser was born in a patch with two high-fecundity females is *α*_0_ = (*u*_0_2(1-*z*_H0_))/(1-$\bar{z}$). The probability that a disperser was born in a patch with one high-fecundity female and one low-fecundity female is *α*_1_ = (*u*_1_((1-*z*_H1_)+(1-*z*_L1_)(1-*s*)))/(1-$\bar{z}$). The probability that a disperser was born in a patch with two low-fecundity females is *α*_2_ = (*u*_2_2(1-*z*_L2_))/(1-$\bar{z}$). The recursion equations are given by

$f_{0}'=P_{f0}\left( \frac{1}{2}\left( \frac{1}{2}+\frac{1}{2}\iota\right)+\frac{1}{2}\iota\right)+\left( 1-P_{f0} \right)\left( \left( \pi_{0}\varphi_{0}+\pi_{2}\varphi_{2} \right)f_{0}+\pi_{1}\varphi_{1}\left( \frac{1}{4}\gamma_{1}+\frac{1}{2}f_{1}+\frac{1}{4}\eta_{1} \right) \right)$, (G6)

$f_{1}'=P_{f1}\left( \frac{1}{2}\left( \frac{1}{2}+\frac{1}{2}\iota\right)+\frac{1}{2}\iota\right)+\left( 1-P_{f1} \right)\left( \left( \pi_{0}\varphi_{0}+\pi_{2}\varphi_{2} \right)f_{0}+\pi_{1}\varphi_{1}\left( \frac{1}{4}\gamma_{1}+\frac{1}{2}f_{1}+\frac{1}{4}\eta_{1} \right) \right)$, (G7)

$\gamma_{1}'=P_{\gamma1}\left( \frac{1}{2}\left( \frac{1}{2}+\frac{1}{2}\iota\right)+\frac{1}{2}\iota\right)+\left( 1-P_{\gamma1} \right)\left( \left( \pi_{0}\varphi_{0}+\pi_{2}\varphi_{2} \right)f_{0}+\pi_{1}\varphi_{1}\left( \frac{1}{4}\gamma_{1}+\frac{1}{2}f_{1}+\frac{1}{4}\eta_{1} \right) \right)$, and (G8)

$\eta_{1}'=P_{\eta1}\left( \frac{1}{2}\left( \frac{1}{2}+\frac{1}{2}\iota\right)+\frac{1}{2}\iota\right)+\left( 1-P_{\eta1} \right)\left( \left( \pi_{0}\varphi_{0}+\pi_{2}\varphi_{2} \right)f_{0}+\pi_{1}\varphi_{1}\left( \frac{1}{4}\gamma_{1}+\frac{1}{2}f_{1}+\frac{1}{4}\eta_{1} \right) \right)$, (G9)

in which the coefficient of inbreeding is given by

$\iota=\left( \pi_{0}\left( 1-h_{0} \right)+\pi_{1}\left( 1-h_{1} \right)+\pi_{2}\left( 1-h_{2} \right) \right)\left( \alpha_{0}f_{0}+\alpha_{1}f_{1}+\alpha_{2}f_{2} \right)+\pi_{0}h_{0}f_{0}+\pi_{1}h_{1}f_{1}+\pi_{2}h_{2}f_{0}$. (G10)

Note that we only need four recursion equations, as the recursion equations for the coefficients of consanguinity *γ*_0_, *η*_0_*, f*_2_, *γ*_2_, and *η*_2_ are all identical to the recursion equation for the coefficient of consanguinity *f*_0_. We find the coefficients of consanguinity by simultaneously solving these recursion equations for equilibrium. The coefficient of consanguinity between a mother and herself is *p* = ½+½*ι.* The coefficient of consanguinity between a mother and her daughter or son is *p*_D_ = *p*_S_ = ½*p*+ ½*ι*. The coefficient of consanguinity between a mother and a daughter or son of the other mother is *p*_F_ = *p*_M_ = (*π*_0_*φ*_0_+ *π*_2_*φ*_2_)*f*_0_+*π*_1_*φ*_1_(½*γ*_1_+½*f*_1_). The relatedness between a mother and her daughters or sons is *r*_D_ = *r*_S_ = *p*_D_ / *p*. The relatedness between a mother and a daughter or son of the other mother is *r*_F_ = *r*_M_ = *p*_F_ / *p*.

*Haplodiploidy* -- Here we focus on haplodipoloid inheritance. In patches with two high-fecundity females and in patches with two low-fecundity females the probability that two juveniles sampled at random are siblings is *P*_f0_ = ½. In mixed patches: (1) the probability that two juveniles of the opposite sex are siblings is given by *P*_f1_ = ((1-*z*_H1_)/(1-*z*_H1_+(1-*z*_L1_)(1-*s*)))(*z*_H1_/( *z*_H1_+*z*_L1_(1-*s*)))+( (1-*z*_L1_)(1-*s*))/(1-*z*_H1_+(1-*z*_L1_)(1-*s*)))( *z*_L1_(1-*s*)/( *z*_H1_+*z*_L1_(1-*s*))); (2) the probability that two female juveniles are siblings is given by *P*_γ1_ = ((1-*z*_H1_)/(1-*z*_H1_+(1-*z*_L1_)(1-*s*)))^2^+((1-*z*_L1_)(1-*s*))/(1-*z*_H1_+(1-*z*_L1_)(1-*s*)))^2^; and (3) the probability that two male juveniles are siblings is given by *P*_η1_ = (*z*_H1_/( *z*_H1_+*z*_L1_(1-*s*)))^2^+( *z*_H1_+*z*_L1_(1-*s*)))^2^. The probability that a female chosen at random after dispersal is native to the patch is given by: (1) *h*_0_ = 2(1-*z*_H0_)(1-*d*)*q*_0_(*z*_H0_,*z*_H0_) in patches with two high-fecundity mothers; *h*_1_ = ((1-*z*_H1_)+(1-*z*_L1_)(1-*s*))(1-*d*)*q*_1_(*z*_H1_,*z*_L1_) in mixed patches; and (3) *h*_2_ = 2(1-*z*_L2_)(1-*d*)*q*_2_(*z*_L2_,*z*_L2_) in patches with two low-fecundity mothers. Thus, the probabilities of co-philopatry are given by *φ*_0_ = *h*_0_^2^, *φ*_1_ = *h*_1_^2^, and *φ*_2_ = *h*_2_^2^. The probability that a focal patch had two high-fecundity females in the previous generation is *π*_0_ = *u*_0_. The probability that a focal patch had a high-fecundity female and a low-fecundity female is *π*_1_ = *u*_1_. The probability that a focal patch had two low-fecundity females in the previous generation is *π*_2_ = *u*_2_. The probability that a disperser was born in a patch with two high-fecundity females is *α*_0_ = (*u*_0_2(1-*z*_H0_))/(1-$\bar{z}$). The probability that a disperser was born in a patch with one high-fecundity female and one low-fecundity females is *α*_1_ = (*u*_1_((1-*z*_H1_)+(1-*z*_L1_)(1-*s*)))/(1-$\bar{z}$). The probability that a disperser was born in a patch with two low-fecundity females is *α*_2_ = (*u*_2_2(1-*z*_L2_))/(1-$\bar{z}$). The recursion equations are given by

$f_{0}'=P_{f0}\left( \frac{1}{2}\left( \frac{1}{2}+\frac{1}{2}\iota\right)+\frac{1}{2}\iota\right)+\left( 1-P_{f0} \right)\left( \left( \pi_{0}\varphi_{0}+\pi_{2}\varphi_{2} \right)\left( \frac{1}{2}{\gamma_{0}+\frac{1}{2}f}_{0} \right)+\pi_{1}\varphi_{1}\left( \frac{1}{2}\gamma_{1}+\frac{1}{2}f_{1} \right) \right)$, (G11)

$\gamma_{0}'=P_{f0}\left( \frac{1}{2}\left( \frac{1}{2}+\frac{1}{2}\iota\right)+\frac{1}{2}\iota\right)+\left( 1-P_{f0} \right)\left( \left( \pi_{0}\varphi_{0}+\pi_{2}\varphi_{2} \right)\left( \frac{1}{4}\gamma_{0}+\frac{1}{2}f_{0}+\frac{1}{4}\eta_{0} \right)+\pi_{1}\varphi_{1}\left( \frac{1}{4}\gamma_{1}+\frac{1}{2}f_{1}+\frac{1}{4}\eta_{1} \right) \right)$, (G12)

$\eta_{0}'=P_{f0}\left( \frac{1}{2}+\frac{1}{2}\iota\right)+\left( 1-P_{f0} \right)\left( \left( \pi_{0}\varphi_{0}+\pi_{2}\varphi_{2} \right)\gamma_{0}+\pi_{1}\varphi_{1}\gamma_{1} \right)$, (G13)

$f_{1}'=P_{f1}\left( \frac{1}{2}\left( \frac{1}{2}+\frac{1}{2}\iota\right)+\frac{1}{2}\iota\right)+\left( 1-P_{f1} \right)\left( \left( \pi_{0}\varphi_{0}+\pi_{2}\varphi_{2} \right)\left( \frac{1}{2}{\gamma_{0}+\frac{1}{2}f}_{0} \right)+\pi_{1}\varphi_{1}\left( \frac{1}{2}\gamma_{1}+\frac{1}{2}f_{1} \right) \right)$, (G14)

$\gamma_{1}'=P_{\gamma1}\left( \frac{1}{2}\left( \frac{1}{2}+\frac{1}{2}\iota\right)+\frac{1}{2}\iota\right)+\left( 1-P_{\gamma1} \right)\left( \left( \pi_{0}\varphi_{0}+\pi_{2}\varphi_{2} \right)\left( \frac{1}{4}\gamma_{0}+\frac{1}{2}f_{0}+\frac{1}{4}\eta_{0} \right)+\pi_{1}\varphi_{1}\left( \frac{1}{4}\gamma_{1}+\frac{1}{2}f_{1}+\frac{1}{4}\eta_{1} \right) \right)$, and (G15)

$\eta_{1}'=P_{\eta1}\left( \frac{1}{2}+\frac{1}{2}\iota\right)+\left( 1-P_{\eta1} \right)\left( \left( \pi_{0}\varphi_{0}+\pi_{2}\varphi_{2} \right)\gamma_{0}+\pi_{1}\varphi_{1}\gamma_{1} \right)$, (G16)

in which the coefficient of inbreeding is given by

$\iota=\left( \pi_{0}\left( 1-h_{0} \right)+\pi_{1}\left( 1-h_{1} \right)+\pi_{2}\left( 1-h_{2} \right) \right)\left( \alpha_{0}f_{0}+\alpha_{1}f_{1}+\alpha_{2}f_{2} \right)+\pi_{0}h_{0}f_{0}+\pi_{1}h_{1}f_{1}+\pi_{2}h_{2}f_{0}$. (G17)

Note that we only need six recursion equations, as the recursion equations for the coefficients of consanguinity *f*_2_, *γ*_2_, and *η*_2_ are all identical to the recursion equation for the coefficient of consanguinity *f*_0_, *γ*_0_, *η*_0_*,* respectively. We find the coefficients of consanguinity by simultaneously solving these recursion equations for equilibrium. The coefficient of consanguinity between a mother and herself is *p* = ½+½*ι.* The coefficient of consanguinity between a mother and her daughter or her son is *p*_D_ = ½*p*+ ½*ι*, whilst the coefficient of consanguinity between a mother and her son is *p*_S_ = ½ + ½*ι*. The coefficient of consanguinity between a mother and a daughter or son of the other mother is *p*_F_ = *p*_M_ = (*π*_0_*φ*_0_+ *π*_2_*φ*_2_)(½*γ*_0_+½*f*_0_)+*π*_1_*φ*_1_(½*γ*_1_+½*f*_1_), whilst the coefficient of consanguinity between a mother and a son of the other mother is *p*_F_ = *p*_M_ = (*π*_0_*φ*_0_+ *π*_2_*φ*_2_)*γ*_0_+*π*_1_*φ*_1_*γ*_1_. The relatedness between a mother and her daughters is *r*_D_ = *p*_D_ / *p*, whilst the relatedness between a mother and her son is *r*_S_ = *p*_S_ / *p*. The relatedness between a mother and the daughters of the other mother is *r*_F_ = *p*_F_ / *p*, whilst the relatedness between a mother and the sons of the other mother is *r*_M_ = *p*_M_ / *p*.

Selection gradient

The selection gradient for the sex ratio expressed conditionally on the mother’s fecundity is given by the slope of her fitness *W* on her breeding value *g* for the sex ratio (Taylor & Frank 1996; Frank 1998). The selection gradients are given by

$\frac{dW_{H0}}{dg_{fH0}}=-c_{f}a_{0}r_{D}+c_{m}\frac{1-z_{H0}}{z_{H0}}a_{0}r_{D}-c_{m}\frac{1}{z_{H0}}\left( u_{H0m}r_{S}+u_{H0m}r_{M} \right)+\left( 1-d \right)q_{0}h_{0}\left( c_{f}\left( u_{H0f}r_{D}+u_{H0f}r_{F} \right)+c_{m}\left( u_{H0m}r_{S}+u_{H0m}r_{M} \right) \right)$, and (G18)

$\frac{{dW}_{H1}}{dg_{fH1}}=-c_{f}a_{1}r_{D}+c_{m}\frac{1-z_{H1}+\left( 1-z_{L1} \right)\left( 1-s \right)}{z_{H1}+z_{L1}\left( 1-s \right)}a_{1}r_{D}-c_{m}\frac{1+\left( 1-s \right)}{z_{H1}+z_{L1}\left( 1-s \right)}\left( u_{H1m}r_{S}+u_{L1m}r_{M} \right)+\left( 1-d \right)q_{1}h_{1}\left( c_{f}\left( u_{H1f}r_{D}+u_{L1f}r_{F} \right)+c_{m}\left( u_{H1m}r_{S}+u_{L1m}r_{M} \right) \right)$, (G19)

$\frac{{dW}_{L1}}{dg_{fL1}}=-c_{f}a_{1}r_{D}+c_{m}\frac{1-z_{H1}+\left( 1-z_{L1} \right)\left( 1-s \right)}{z_{H1}+z_{L1}\left( 1-s \right)}a_{1}r_{D}-c_{m}\frac{1+\left( 1-s \right)}{z_{H1}+z_{L1}\left( 1-s \right)}\left( u_{H1m}r_{M}+u_{L1m}r_{S} \right)+\left( 1-d \right)q_{1}h_{1}\left( c_{f}\left( u_{H1f}r_{F}+u_{L1f}r_{D} \right)+c_{m}\left( u_{H1m}r_{M}+u_{L1m}r_{S} \right) \right)$, and (G20)

$\frac{dW_{L2}}{{dg}_{fL2}}=-c_{f}a_{2}r_{D}+c_{m}\frac{1-z_{L2}}{z_{L2}}a_{2}r_{D}-c_{m}\frac{1}{z_{L2}}\left( u_{L2m}r_{S}+u_{L2m}r_{M} \right)+\left( 1-d \right)q_{2}h_{2}\left( c_{f}\left( u_{L2f}r_{D}+u_{L2f}r_{F} \right)+c_{m}\left( u_{L2m}r_{S}+u_{L2m}r_{M} \right) \right)$, (G21)

Under self-knowledge females know their own fecundity but not that of their patch mates. Therefore we set *z*_H0_ = *z*_H1_ = *z*_H_, and *z*_L1_ = *z*_L2_ = *z*_L_. The selection gradient of high-fecundity mothers is then given by *S*_H_ = *u*_H0_(d*W*_H0_/d*g*_fH0_) + *u*_H1_(d*W*_H1_/d*g*_fH1_), while the selection gradient of low-fecundity mothers is given by *S*_L_ = *u*_L1_(d*W*_L1_/d*g*_fL1_) + *u*_L2_(d*W*_L2_/d*g*_fL2_). To determine the optimal sex allocation strategy, we set these selection gradients to zero (i.e. *S*_H_ = 0 and *S*_L_ = 0), and we solve the system of equations for equilibrium.

**Appendix H. Supplementary figures**

**
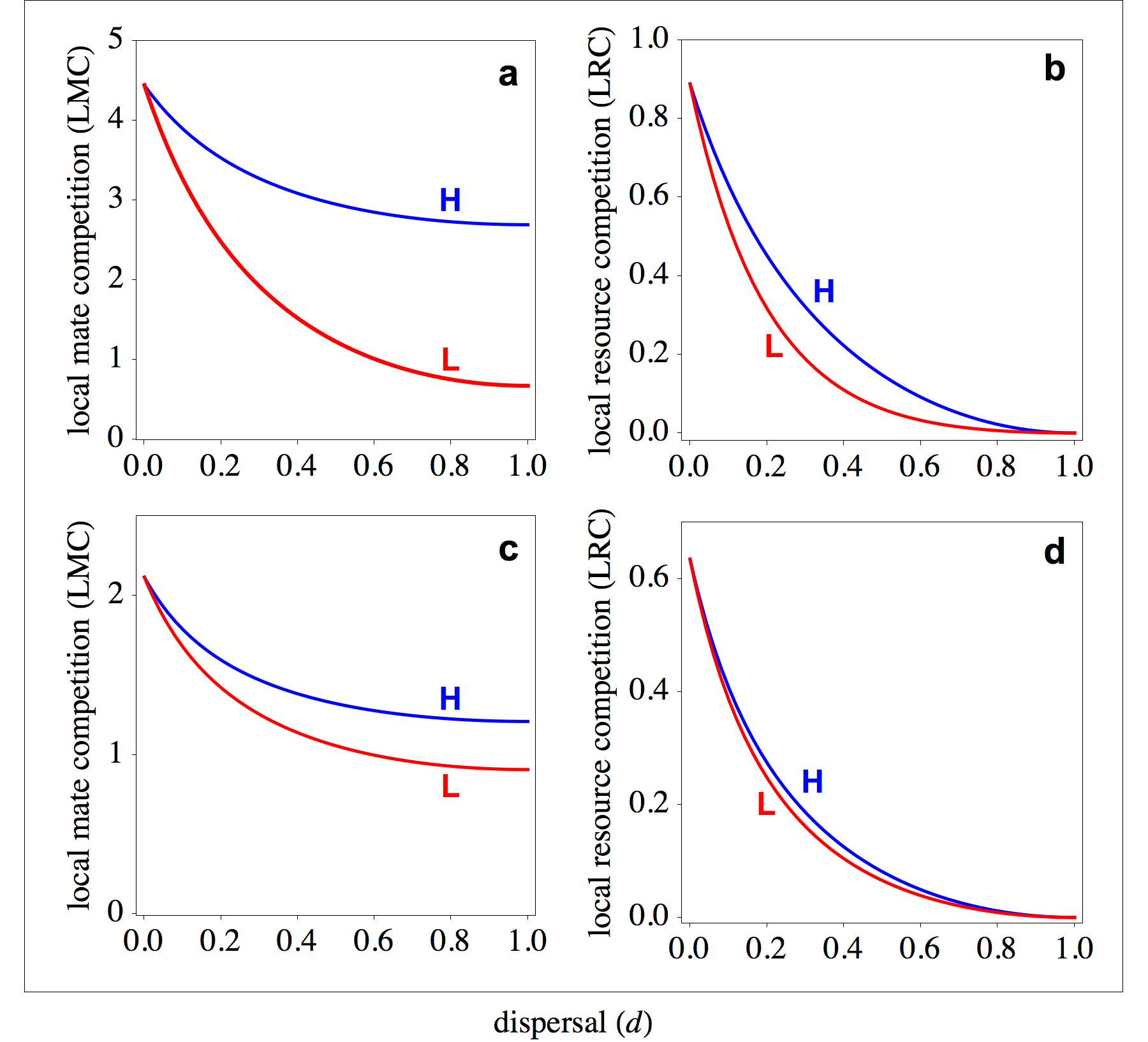
**

**Figure H1 | Local mate competition and local resource competition in viscous populations.** The strength of local mate competition (given by the third term in the selection gradients) and the strength of local resource competition (given by the fourth term in the selection gradients) for high- and low-fecundity mothers as a function of the dispersal rate (*d*) for haploidy and diploidy (panels (a) and (b)) and for haplodiploidy (panels (c) and (d)), assuming the parameter values *k* = 0, *s* = 0.75, *z*_H_ = *z*_L_ = *z* = 0.1.


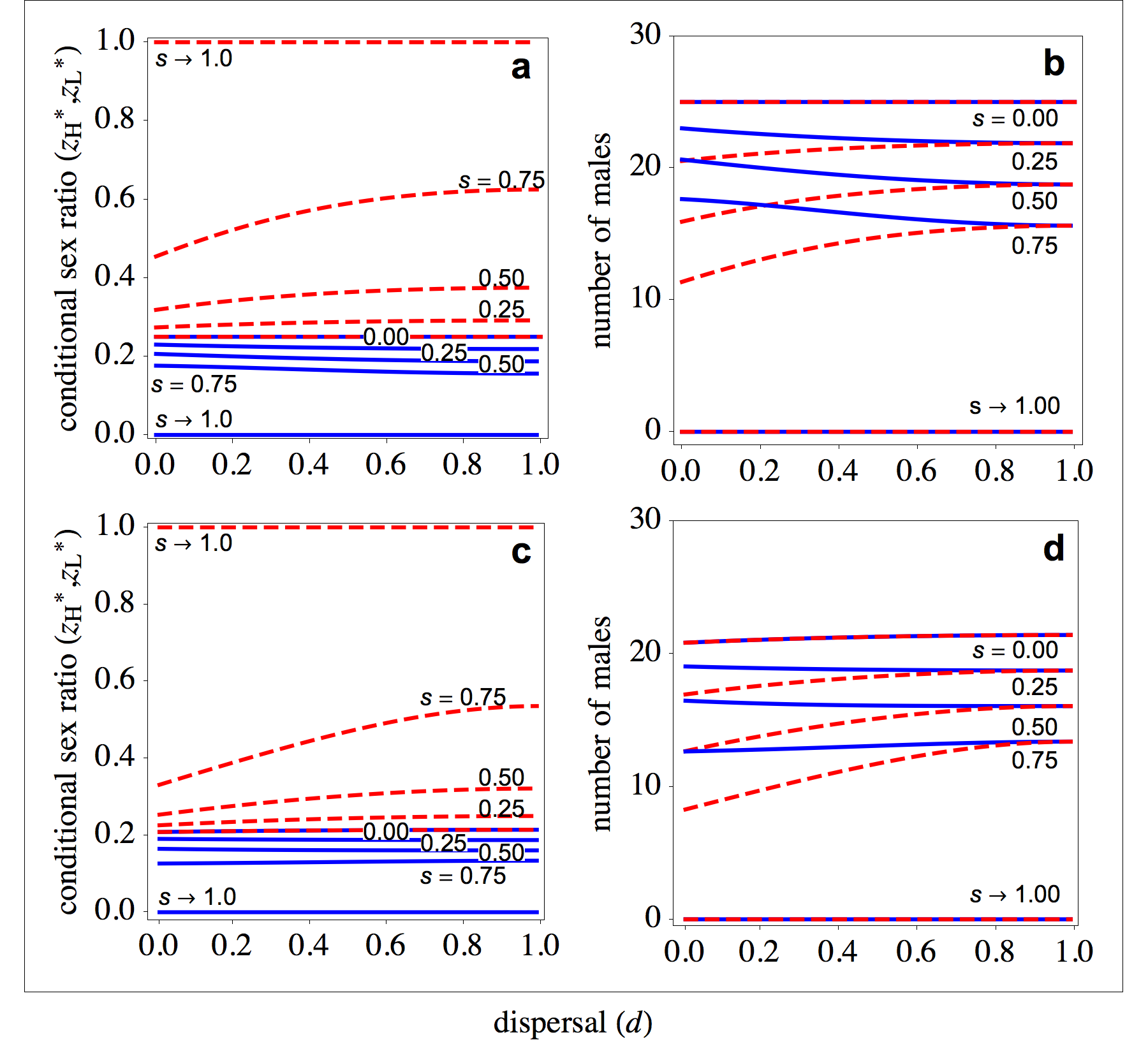


**Figure H2 | Facultative sex allocation.** (a,b) High-fecundity mothers (blue lines) are favoured to invest relatively more into sons than are low-fecundity mothers (red dashed line) in viscous populations (*d* < 1), under haploidy and diploidy. (c,d) High-fecundity mothers (blue line) are favoured to invest relatively more into sons than are low-fecundity mothers (red dashed lines) in viscous populations (*d* < 1), though both are favoured to invest less into sons, under haplodiploidy. We arbitrarily set the total number of offspring of a high-fecundity mother to 100. Parameter values: *k* = 0.


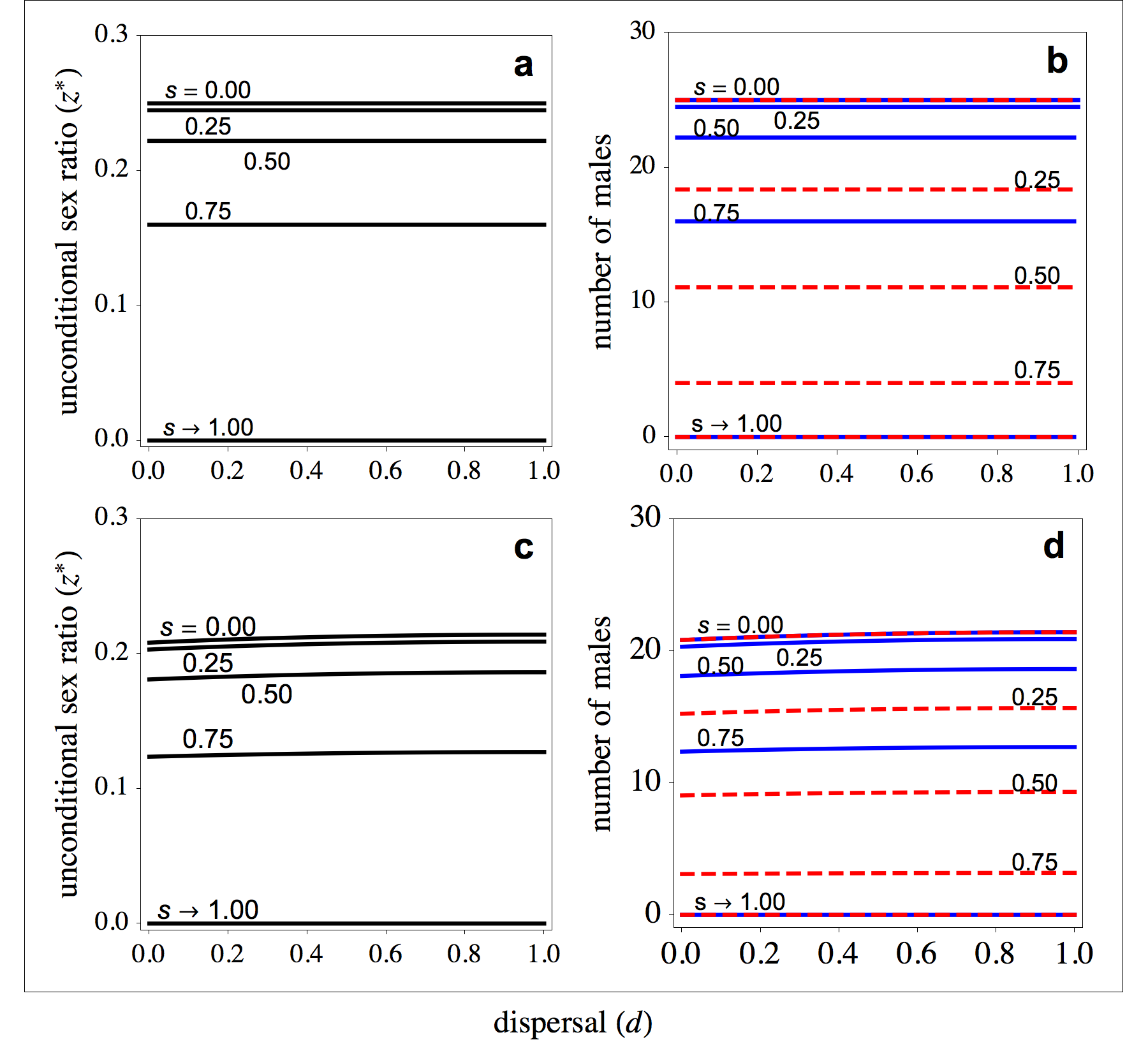


**Figure H3 | Obligate sex allocation.** (a,b) If mothers are obliged to invest a fixed amount into sons, irrespective of their fecundity, then their investment into sons is independent of the degree of viscosity, under haploidy and diploidy. (c,d) If mothers are obliged to invest a fixed amount into sons, irrespective of their fecundity, then their investment into sons slightly decreases as populations become increasingly viscous (lower *d*), under haplodiploidy. As both mothers invest the same proportion of resources into males, but differ in the absolute amount of resources they have, high-fecundity mothers give birth to more sons (blue lines) than low-fecundity mother (red dashed lines). We arbitrarily set the total number of offspring of a high-fecundity mother to 100. Parameter values: *k* = 0.

**References**

Bulmer, M.G. 1994. *Theoretical evolutionary ecology*. Sinauer, Sunderland, Massachusetts.

Christiansen, F. B. 1991. On conditions for evolutionary stability for a continuously varying character. *Am*. *Nat*. **138**, 37–50.

Eshel, I. 1996. On the changing concept of evolutionary population stability as a reflection of a changing point of view in the quantitative theory of evolution. *J. Math. Biol.* **34**, 485–510. (doi:10.1007/BF02409747)

Frank, S. A. 1998. *Foundations of social evolution*. Princeton Univ. Press, Princeton, NJ.

Taylor, P. D. 1996 Inclusive fitness arguments in genetic models of behaviour. *J Math Biol* **34**, 654–674. (doi:10.1007/BF02409753)

Taylor, P. D. & Frank, S. A. 1996 How to make a kin selection model. *J. Theor. Biol.* **180**, 27–37. (doi:10.1006/jtbi.1996.0075)
